# Assessing incentives to increase digital payment acceptance and usage: A machine learning approach

**DOI:** 10.1371/journal.pone.0276203

**Published:** 2022-11-02

**Authors:** Jeff Allen, Santiago Carbo-Valverde, Sujit Chakravorti, Francisco Rodriguez-Fernandez, Oya Pinar Ardic

**Affiliations:** 1 World Bank, Washington, DC, United States of America; 2 University of Granada, Granada, Spain; 3 Chakra Advisors LLC, Washington, DC, United States of America; URV: Universitat Rovira i Virgili, SPAIN

## Abstract

An important step to achieve greater financial inclusion is to increase the acceptance and usage of digital payments. Although consumer adoption of digital payments has improved dramatically globally, the acceptance and usage of digital payments for micro, small, and medium-sized retailers (MSMRs) remain challenging. Using random forest estimation, we identify 14 key predictors out of 190 variables with the largest predictive power for MSMR adoption and usage of digital payments. Using conditional inference trees, we study the importance of sequencing and interactions of various factors such as public policy initiatives, technological advancements, and private sector incentives. We find that in countries with low POS terminal adoption, killer applications such as mobile phone payment apps increase the likelihood of P2B digital transactions. We also find the likelihood of digital P2B payments at MSMRs increases when MSMRs pay their employees and suppliers digitally. The level of ownership of basic financial accounts by consumers and the size of the shadow economy are also important predictors of greater adoption and usage of digital payments. Using causal forest estimation, we find a positive and economically significant marginal effect for merchant and consumer fiscal incentives on POS terminal adoption on average. When countries implement financial inclusion initiatives, POS terminal adoption increases significantly and MSMRs’ share of P2B digital payments also increases. Merchant and consumer fiscal incentives also increase MSMRs’ share of P2B electronic payments.

## 1 Introduction

In 2013, the World Bank Group President announced the Universal Financial Access (UFA) goal, which stresses that adults globally should have access to a transaction account to safely store money, send and receive payments as the basic building block to manage their financial lives. In 2016, Committee on Payments and Market Infrastructures (CPMI) and the World Bank [1] provided a framework to achieve UFA.

[2] further elaborated on payments serving as a gateway to other financial services. As individuals make several payments daily to purchase food items and other basic needs, generally, the use of payment services is the first time an individual can potentially be introduced to the regulated financial sector. Based on their needs to mitigate risks and invest for the future, individuals can then start using other financial services such as savings, insurance and credit, typically tailored to their needs, ideally delivered responsibly and sustainably in an affordable way. Financial inclusion has been recognized as an important policy goal internationally for more than a decade.

Global Findex 2017 data, which is used to measure progress towards UFA, reports that account ownership at a regulated institution has increased: in the six-year period between 2011–2017, there were 1.2 billion new accountholders globally. Despite greater transaction account ownership, challenges remain as 1.7 billion adults lack access to a basic transaction account. Progress has also been uneven: globally, 72 percent of men and 65 percent of women have an account—reflecting a gender gap that has remained unchanged since 2011 and 2014. Additionally, the gap between the richer 60 percent and the poorer 40 percent, in terms of account ownership, has remained the same throughout the period. The data shows that the unbanked are predominantly women, poor, not well-educated and unemployed. Importantly, nonbanks are increasingly playing a larger role in the provision of transaction accounts by increasing access to the unbanked.

[3] provide empirical evidence regarding the benefits of digital payments including safety, quicker receipt of payments, and lower costs. Global Findex database also tracks how frequently transaction accounts are used to make digital payments. The indicator “made or received digital payments” is calculated based on one payment made or received with the account owned. In 2017, 52 percent of adults globally made or received digital payments as opposed to 42 percent in 2014. Finally, the findings of Global Findex 2017 indicate that a significant proportion of accounts remain inactive for at least 12 months. Globally, one in five account owners had an inactive account during the past 12 months, while in India this figure was as high as one in two.

These findings suggest that policies that solely increase transaction account ownership are not sufficient to increase usage of digital payments. Account ownership must be coupled with greater opportunities for account owners to use their transaction accounts for making and receiving digital payments. Account owners need to have opportunities to use their digital payment instruments for everyday purchases, bill payments, online payments, and government payments. In addition, they would also benefit from digitally receiving their salaries and payments from government, businesses, and other individuals.

In this paper, we assess the effectiveness of various public and private sector initiatives including financial inclusion, better tax collection, and adoption of new technologies on digital payment acceptance by micro, small, and medium retailers (MSMRs) and the increase in the number of digital payments. In 2016, the World Bank commissioned a study to analyze the size of cash versus digital transactions made and received by MSMRs to better understand whether there are sufficient opportunities for consumers to use their transaction accounts to make payments at small, everyday merchants [1]. This issue was considered important because it is through these everyday merchants that retail payment solutions become valuable to consumers and use of electronic payment instruments become habitual, generating an anchor for them in the regulated financial sector. While MSMRs’ acceptance of digital payments is important for consumers, it is also important for these merchants to be able to make digital payments in the form of salaries or supplier payments. Therefore, the World Bank study also looked at business-to-business (B2B –in the form of immediate supplier payments) and business-to-person (B2P) payments. In other words, achieving greater digital liquidity is the goal of many policymakers. The study estimated that MSMRs globally made and accepted USD 34 trillion worth of payments annually, of which only 44 percent were made digitally suggesting that there is a USD 19 trillion opportunity.

We use machine learning techniques to evaluate the effectiveness of public and private sector initiatives along with technological advancements to increase the acceptance of digital payments by MSMRs and to increase the volume of digital payments at the point of sale (POS). We identify key conditions and incentives that predict acceptance and usage of digital payments by MSMRs. Importantly, the order of implementation (or a specific combination) of conditions and the types of incentives matter. With large amounts of data and of covariates, we can only achieve this sequencing using machine learning. Furthermore, we do not limit our analysis to merchant incentives but also consider incentives given to other participants in the payment ecosystem along with improvements to the payment and telecommunication infrastructures that promote digital payment adoption and usage.

Our dependent variables are POS terminal adoption by MSMRs needed for payment card acceptance and the share of digital payments made to MSMRs by individuals. [4] use POS terminals to study the impact of payment cards on transactional cash demand in advanced economies. They find that POS terminal adoption does decrease transactional demand. However, in emerging and developing economies, bank account ownership is not always common. Unfortunately, POS terminal adoption only captures the acceptance of payment cards. However, the volume of digital payments captures all forms of digital payments. While the volume of digital payments does not directly capture the adoption of acceptance infrastructure, it does indirectly capture the adoption of merchants of digital payments beyond card payments. Given the large number of covariates in our cross-country dataset, we use machine learning techniques to identify strong predictors without imposing a structure on the data as is the case for partial or structural standard econometric models. We analyze 81 country-level variables from 106 countries and 111 merchant-level variables for 576 merchants across seven countries. Using conditional inference trees, we identify combinations of predictors that increase the likelihood of increased acceptance and usage of digital payments. Finally, using causal forest estimation, we quantify the impact of different incentives on acceptance and usage of digital payments by comparing countries that have implemented to those that did not.

Our main results are as follows. Using random forest estimation techniques, we identify several factors that are strong predictors for greater POS terminal adoption and higher shares of person-to-business (P2B) electronic payments at MSMRs such as information and communication technologies (ICT) infrastructure, level of transaction account ownership in the economy, fiscal incentives, the size of the shadow economy, digitization of payment chain, and introduction of “killer applications.” In our sample, a killer application is defined as a successful mobile application that enables digital payments.

Using conditional inference trees, we are able to study these factors and predict a sequence of incentives or initiatives that enables greater acceptance and usage of digital payments. For example, if a country is above the median in ICT infrastructure and killer applications are implemented or the proportion of payment service agents are high, the share of P2B MSMR digital payments are predicted to be up to 60 percent higher.

Using causal forest estimation, we consider incentive implementation in different countries as a quasi-natural experiment. While some caveats should be considered as this is not a purely randomized experiment and, therefore, our results cannot be directly extrapolated to experiences in jurisdictions not considered in our sample, the estimated treatment effects reinforce the idea that incentives to electronic payment acceptance (EPA) may have significant economic effects. In our setting, the treatment effects consisting in evaluating a number of country-level policies at merchant level. Some of these policies (e.g. cash limits) are applied in various countries while others are just applied in specific countries. Hence, our approach is connected to a strand of research that uses causal forest to evaluate cross-country public policies, while the impact is evaluated at the individual level. In particular, we find that fiscal incentives, killer applications along with government policies to limit cash transactions or mandate digital payments for certain types of transactions are effective to increase merchant acceptance and usage of digital payments. In addition, we find that when killer applications are introduced in countries that do not have mass adoption and usage of payment cards, mobile payments are able to leapfrog payment cards as the dominant digital payment instrument at the POS.

This article is structured as follows. In the next section, we discuss the current literature on payment merchant acceptance and usage. In section 3, we discuss the data and our empirical approach. In section 4, we discuss our results. In section 5, we discuss the policy implications of our findings. In section 6, we offer some conclusions. Section 7 discusses some limitations of the study.

## 2 Literature review

Our paper contributes to various strands of the payments and non-payments literatures. Given that the market for payments has two distinct end-users—consumers and merchants—payment networks or platforms need to bring both sides on board for a transaction to occur. This aspect of payment services is often referred to as a two-sided market [5, 6]. Generally, incentives on the consumer side have been greater than the merchant side, however incentives to merchants continue to increase especially for MSMRs.

For the most part, this literature focused on payment cards. We extend the two-sided market literature by looking at much broader policy levers than regulating payment card fees, the structure of card networks, and the ability of merchants to pass on payment costs to consumers by imposing surcharges or discounts based on the payment instrument used.

As discussed, consumer access to transaction accounts and digital payment instruments continues to increase around the world. Several empirical studies focus on the how consumers choose to use digital payments instead of cash and checks [7–9]. [10] find that transaction size and consumer demographic factors determine how consumers pay at grocery stores. In some countries, payment platforms bundle goods and services to encourage digital payments at the POS, e.g. Alipay and WeChat Pay in China [11].

Empirical research on the merchant side is more limited. As noted by [12] most studies have ignored or only tangentially consider merchant acceptance. These authors stress the interactions between acceptance and demand. They highlight the challenges for payment platforms to attract critical mass of consumers and merchants. However, theoretical research on why merchants accept payment cards has been growing including the ability of merchants to increase sales to cash and credit-constrained customers and reduce the costs of safekeeping and transporting cash [13, 14].

There have been some payment adoption and usage studies that analyze how incentives affect both consumer and merchant usage of digital payments. Some of these incentives include merchants to steering consumers with differentiated prices based on payment instrument used or by payment service providers offering usage rewards such as cash back. For example, [15] reviews some evidence that supports consumers responding more to merchant surcharges than to discounts depending on the payment instrument used. [16] also examine how U.S. merchants used their ability to offer price discounts and other incentives to steer customers to pay with methods that are less costly to merchants. While not as common as before, some payment networks do not allow merchants to surcharge their branded payment instruments. Various studies have also looked at the impact of card rewards issued by issuers to increase card payments especially for credit card purchases [17, 18].

Other consumer steering incentives have been related to the adoption of mobile payment technology. For example, the case of rewards and/or cash back offered by card companies as well as other NFC payment providers (Apple Pay, Samsung Pay, and Android Pay) for adoption and usage of mobile payments [19] find that these incentives have a positive effect on the decision to adopt NFC mobile payments. While these and other similar incentives may provide some interesting insights, more evidence would be needed on wider (public policy or private-public partnerships) attempts to increase merchant acceptance and consumer usage. Our study also attempts to complement this wider view on incentives especially merchant incentives.

We also contribute to the empirical literature on technology diffusion of new payment technologies. Technology has lowered costs, increased access, and expanded features. Mobile phone technology can be leveraged to make payments. Mobile phone technology along with quick response (QR) codes significantly reduce the cost of payment acceptance from traditional payment card technologies. In some cases, these technologies allow consumers and merchants to adopt mobile payments instead of payment cards. [20] argue that mobile payments can be card-complementing or card-substituting. In countries with high adoption of payment cards and card acceptance infrastructure, mobile payments complement cards whereas in countries with low adoption of cards, mobile payments substitute for card transactions. [20] construct a model where payment technologies arrive sequentially. Under certain cost assumptions, using simple empirical analysis, they explain why advanced economies have not embraced mobile payments unlike developing countries.

Additional incentives may be necessary to reduce the reluctance to use digital payments that are not due to the direct cost of acceptance. The reluctance to use digital payments may be due to non-payments related reasons such as tax evasion. Using a merchant survey of Indian small merchants in Jaipur, [21] considers other factors that prevent adoption of digital payments by merchants, including demand-side factors and taxes. Some researchers have also investigated a more direct intervention such as restricting cash use to reduce the size of the shadow economy or mandating electronic payments for certain types of transactions [22, 23].

Finally, recent academic studies suggest that structural, as well as policy-related factors, such as channeling government benefit payments through transaction accounts, play an important role in improving financial inclusion [24]. Account ownership is not only critical to access formal payments [25], it also reduces corruption [26], and increases consumption and productive investment of entrepreneurs [27]. Additionally, shifting payments from cash to those using transaction accounts allows for more transparent and efficient payments especially for payments between individuals and governments [3]. Government interventions such as demonetization intended to reduce the shadow economy may have had positive implications for adoption for digital payments [28].

## 3 Empirical methodology and data

In this section, we describe our empirical approach and our dataset. We use machine learning algorithms to identify predictors, provide insights into sequencing and interactions of various incentives, and quantifying treatment effects of individual incentives. We also describe how we combine different sources to create our dataset.

### 3.1 Empirical aim and approach

Most existing studies in the area of digital financial services use discrete choice models to examine consumers’ preferences for various types of payments and other financial services [29–31]. However, recent studies have shown that capturing changes in behavior from traditional choices to digital options involves several factors, including socio-economic, behavioral and institutional characteristics [32]. These multifaceted patterns suggest that a multidisciplinary approach combining economic, behavioral and data analytics is required to address such changes [33]. Machine learning methods are powerful data analysis tools that hold promise for generating new insights into payment behavior [34–36]. [37] surveys a number of methods used in studies of changes in demand behavior and concludes that machine learning techniques are effective and, often, more powerful for this type of analysis.

The advantages of a machine learning approach where there are many potential influences at the macroeconomic and microeconomic levels, such as digital payments, has increased its use for such analysis. For example, [38] uses a random forest model to discover hidden patterns that may be valuable for decision-making in bank marketing. Machine learning approaches are also employed to estimate consumer preferences for technology products [39], to examine travel choices [40], and, more generally, to model consumer response [34].

Building on this growing literature, our empirical analysis employs machine learning techniques to analyze a combination of payment ecosystem (macro) and payment system participant specific (micro) factors. Payment ecosystem factors include improvements to payment and nonpayment infrastructure, national ID programs, technological advancements in communication and computing technologies, and policies to reduce the shadow economy. Payment system participant factors include government policies to increase transaction account ownership, payment service provider (PSP) incentives, fiscal incentives to consumers and merchants to adopt digital payments, and technological advances that allow PSPs to provide payment services to more consumers and businesses often at lower costs that impact electronic payment acceptance (EPA) and usage. These incentives are defined in line with previous World Bank research [41].

The machine learning approaches that we employ carry three primary advantages over traditional parametric statistical methods. First, machine learning is a data-driven, bottom-up analytical approach or commonly referred to as “let the data speak” approach. It requires no preestablished or strict assumptions regarding the structure of the data or the functional relationships. Second, because we rely on tree-based machine learning mechanisms, our analysis may offer some insights on combinations of incentives and decision sequencing. While standard parametric models in econometrics (e.g. multilevel logit and propensity score matching) can identify behavioral patterns among clusters of individuals of firms, they are unable to offer predictions based on a sequence of actions. In other words, for some of the results obtained, the order of implementation (or a specific combination) of factors matters. In our context, previous empirical analyses and the policy experience in several countries seem to suggest that some actions or conditions can only be effective in promoting merchant EPA when other actions and conditions are already in place. For example, technology such as mobile phones or QR codes or payment infrastructure such as fast payment or card networks as a pre-condition for some payments to be accepted. However, when the number of actions and conditions is large (as in our setting) machine learning can be used to try to find sequences that increase EPA. Taken together, these first two advantages yield an analytical approach that uncovers patterns in real world interactions that induces greater adoption of merchant digital payment acceptance infrastructure and digital payment usage than what could be captured with a top-down statistical model that imposes a global structure on the data. Such an approach is also important to extract policy lessons for further merchant acceptance of electronic payments in countries with current low levels of acceptance as the global treatment of the data somehow elucidates a number of benchmarks or paths for these countries to follow. Given the treatment of data in our machine learning approach, it would not make much statistical sense to conduct specific sub-sample analyses for a smaller set of countries (e.g., low-income countries) as variability within a single group would not provide much information on benchmarking and potential routes for merchant acceptance development. Lastly, from a predictive standpoint, our machine learning approaches are more accurate than traditional econometric models used in the banking and payments literature.

However, the primary disadvantage of machine learning is that it typically emphasizes prediction over inference. Economists have traditionally been interested in studying what factors cause a change in a dependent variable as opposed to what factors predict the movement in a dependent variable. In our analysis, we are not only interested in what factors or preconditions predict digital payment acceptance and usage but also what incentives would change the behavior of merchants and consumers to increase their usage and adoption of digital payments. We partially overcome this limitation primarily because of data limitations by utilizing three complementary machine learning models, the respective features of which allow us to draw inferences about the drivers of digital payment acceptance. Ideally, we would like to use a panel dataset where we could also study adoption over time within a country. Also as mentioned above, we would prefer greater merchant heterogeneity across the incentives that we study.

Specifically, our analysis follows a three-step process. In the first step, we use the random forest algorithm [42] to analyze and narrow down the list of variables that are most important for predicting our dependent variables. For digital payment acceptance, we use POS terminal adoption because merchants generally must have POS terminals to accept payment cards. Unfortunately, we do not have reliable data for non-card merchant acceptance infrastructure. To account for the growth of non-card-based payments, we also use MSMRs’ share of P2B digital payments. While not a direct approach, the share of digital payments also captures the adoption of payment infrastructure because acceptance infrastructure must be in place before payments can be made. In addition, share of digital payments allows us to capture the usage of digital payments which is the eventual goal of policymakers and digital PSPs.

In the second step, we use the most important variables identified by the random forests to build conditional inference trees [43], which more specifically identify the sequential paths of adoption. In other words, we identify the likelihood of greater POS terminal adoption and usage based on a combination or a sequence of factors. These conditional inference trees isolate important sets of factors for prediction.

In the last step, we leverage the recently developed causal forest [44] and [45]—an adaptation of random forests for more inferential purposes—to estimate treatment effects of relevant incentives on payment acceptance. Causal forests are frequently implemented as randomized experiments in a controlled sample. In these cases, causal forests may assist to sort the information and calibrate the average treatment effects when the number of covariates is large. For example, [46] explores the impact of a randomized implementation of a microcredit program on the money borrowed and pension plan eligibility. While randomized experiments may be applied in the assessment of public policies, they cannot always be implemented. This occurs when the experimentation protocol is too expensive or complex. However, causal forests can also be used (with the necessary interpretation caveats) in the context of quasi-natural experiments. In particular, if some policies are exogenously implemented and identified as a potential shock or driver of change of certain behavior. We study the effects of groups that are treated or receive a type of incentive to increase EPA versus groups that do not receive that incentive. For the country-level sample, we study the impact of incentives on a treated country and on an untreated country. For the merchant-level sample, we study the impact on merchants within a treated country and merchants in an untreated country (see [47], on the use of causal forest to evaluate policies at the country-level).

In exploring the mechanisms underlying digital payment acceptance and usage, we take a general-to-specific approach. That is, we first analyze a “general mechanism,” that consists of identifying and rank the predicting power of country-level indicators to identify incentive mechanisms broadly. In particular, we strive to explain the absolute or conditional weight of some factors on the success of digital payment acceptance and usage incentives. This exercise provides a general framework with respect to successful implementation steps for different incentives. From a data perspective, the country level data allows us to study 106 countries rather than only the seven countries that we have merchant survey data for.

Second, in analyzing a merchant-level sample, we explore a more “detailed mechanism,” which describes the individual path followed by merchants in accepting various forms of payments and identifies the role of some incentives specific to merchants. As with the country-level analysis, the merchant level analysis investigates the effects of incentives on EPA. The following subsections expand on the data, we use the three sequential analytical approaches that we employ—the random forest, the conditional inference tree, and the causal forest.

### 3.2 Data

In this subsection, we describe how we construct our dataset. We combine a cross-section of country level explanatory variables with merchant survey data to identify and quantify the impact of incentives on electronic payment acceptance (EPA) infrastructure and usage.

#### 3.2.1 Country-level data

The country-level data are cross-sectional and mostly from 2014 with some exceptions because data was not available for 2014. However, we do not believe these differences will affect the identification of merchant EPA. While our primary response variables for both the country-level and merchant-level data are POS terminal adoption and MSMRs share of digital payments, they are measured differently. At the country-level, we use POS terminals per 100,000 adults at year-end 2014, which we obtain from the World Bank’s Global Payment System Survey (GPSS). As mentioned before, a natural limitation of modeling POS terminals is they focus primarily on card-based payments and do not capture the emerging non-card-based methods of electronic payments. P2B electronic payments, however, do capture all forms of electronic payments. For MSMRs’ share of P2B digital payments at the country-level, we use a country’s estimated MSMRs’ share of digital payments. [1] (hereafter, the “Sizing Study”) estimated the MSMRs’ share of digital payments for mid-to-late 2015.

As discussed further below, the Sizing Study is based on primary research in seven representative countries. The research included in-person trade interviews and pulse surveys to complement existing research and data collection by Euromonitor International. Detailed information on this survey is provided in [1]. The trade interviews, which were conducted with government agencies, retail associations, financial institutions, and non-bank financial service providers, provide a top-down perspective of the retail payments market. The pulse surveys, which were conducted with individual retailers and suppliers, provide a bottom-up perspective of the retail payments market. Information from both sources were consolidated to produce country-level estimates for the value and volume of P2B, business-to-business (B2B), and business-to-person (B2P) payments by MSMRs such as paying workers. In our dataset, B2B payments include immediate supplier payments only, and not the entire supply chain. The estimates for the seven representative countries were paired by Euromonitor with selected assumptions and other macroeconomic and financial data to estimate these variables for 161 other countries using simulation methods. Simulations of the Sizing Study conducted by Euromonitor are explained in Annex A4.3 of [1]. Several models were used for the simulation of the main variables. The 30 best performing models based on R2 were used to predict an outcome variable. Then, these 30 models are evaluated for the out-of-sample fit. The predictions from these 30 models for one outcome variable are then averaged to construct the final prediction for that outcome variable, thereby relying on out-of-sample predictions of the best-fitting 30 models for each of the 61 variables to be predicted.

As already indicated, the country-level share of electronic P2B payments from the Sizing Study serves as one of our two primary country-level response variables. We use the B2B and B2P electronic payment estimations as predictor variables. We consider the whole MSMR payment chain because it has important implications for incentive design and effectiveness. In particular, incentives may encourage consumers to use bank accounts rather than cash alternatives. Digital payment acceptance by MSMRs is a critical factor to allow consumers to use digital payments. At the same time, MSMRs may also benefit from incorporating digital payment infrastructure to pay salaries and suppliers digitally. Hence, policies such as mandated digital payments for MSMR B2B and B2P payments can be strongly connected to P2B digital payments.

The other country-level predictor variables are drawn from various sources. The aim is to obtain as much information as possible on potential determinants of digital payment acceptance and usage to feed the random forest algorithm and ultimately narrow down a core group of predictors. With some exceptions, most of our observations are from 2014, corresponding with the Sizing Study timeframe. A first reference for these variables is the GPSS, which surveys national and regional central banks and monetary authorities on the status of payment system development (e.g., e-money accounts per 1,000 adults and agents of payment service providers per 100,000 adults).

A number of variables are also drawn from the World Bank’s Global Findex database. The database collects information on how adults save, borrow, make payments, and manage risk through nationally representative surveys of more than 150,000 adults in over 140 economies. The data employed are primarily from 2014. We also use the 2011 Findex database to compute the change from 2011 to 2014 for some variables (in particular, those reflecting payment usage).

A third database that we use in our analysis is the Global Financial Inclusion and Consumer Protection (FICP) Survey from the World Bank, which tracks the prevalence of key policy, legal, regulatory, and supervisory approaches for advancing financial inclusion and consumer protection, including national financial inclusion strategies, the issuance of e-money by nonbanks, agent-based delivery models, simplified customer due diligence, institutional arrangements for financial consumer protection, disclosure, dispute resolution, and financial capability. Financial sector authorities in 124 jurisdictions—representing 141 economies and more than 90 percent of the world’s unbanked adult population—responded to the survey. The data were not available for 2014 and the closest date that they were available was 2017. We believe these variables are quite structural and the time difference does not affect our results. See S1 Appendix for a detailed reference on the year corresponding to each variable.

We also use data from the Financial Access Survey (FAS) compiled by the International Monetary Fund (IMF). The FAS provides data on access to and use of financial services aimed at supporting policymakers to measure and monitor financial inclusion and benchmark progress against peers. FAS covers 189 countries spanning more than 10 years and contains 121 time-series on financial access and use. We use FAS 2014 data in our analysis.

We also use two other databases for very specific information on information technology development (the ICT Development Index, IDI, provided by the United Nations’ International Telecommunications Union, ITU) and crime level information (from the United Nations Office for Drug and Crime, UNDOC).

In the end, 81 variables are selected for 106 countries, resulting in 8,586 cross-section datapoints. S1 Appendix provides the list of countries and variable definitions.

#### 3.2.2 Merchant-level data

Merchant-level data is also cross-sectional and allows us to study merchant heterogeneity within MSMRs while allowing for some country heterogeneity. The merchant-level data is based on the Sizing Study primary research pulse surveys, which are introduced above. Primary research was conducted in seven countries in 2015—Colombia, France, Kenya, Lithuania, Morocco, Pakistan, and Turkey. Similar to our constructed country-level dataset, our two response variables capture POS adoption and P2B electronic payments. However, because these are merchant-level indicators, they take on different forms from the country-level data. The POS variable is a binary (0/1) indicator capturing whether an individual merchant has a POS terminal or not. The P2B electronic payments indicator captures the estimated share of P2B payments made electronically at the individual retail establishment.

The Sizing Study database contains rich retailer-level data, which we use as predictor variables. These include indicators such as retailer size, customer profiles, merchant and consumer preferences, and whether retailers are part of a larger network, among other characteristics. As with the country-level data, we also use retailer-level data on electronic B2B and B2P payments as predictors. Further, we combine the merchant-level information with a number of country-level indicators similar to those employed in the country-level sample. In total, the merchant-level database consists of 576 merchants and 111 variables, resulting in 63,936 cross-section and time series datapoints. All the variables are defined in S1 Appendix. The number of variables in the appendix could be lower as some merchant-level variables are aggregated at the national level to be used in the country-level analysis and some national-level variables are used as control variables in the merchant level analysis. While the merchant-level data corresponds to 2015 and the country-level data to 2014 we believe a one-year difference is not relevant for our empirical analysis.

We use the merchant-level data for two primary empirical purposes. First, we focus on the detailed merchant-level information for the seven countries to check if the machine learning results from the microeconomic structure are similar to those obtained at the country level. Second, we add information on policy incentives for the seven countries to analyze their impact on digital payment acceptance and usage.

In matching the different sources of information for the country-level and merchant-level data, we were particularly careful in using homogenous measures. In order to make the economic interpretations of the results more tractable and, at the same time, ensuring sufficient heterogeneity within the country- and merchant-level data, all quantitative variables in the database were transformed into four-level variables with values 1, 2, 3 and 4 corresponding to percentiles 0-25^th^ (low), 25th-50^th^ (lower mid), 50th-75^th^ (upper mid) and 75th-100 (high). Additionally, a number of variables contained some missing or not available (NA) values for some observations. However, the different algorithms employed in the machine learning techniques deal well with it treating them as missing values.

### 3.3 Identifying variable importance using random forest

The random forest [42] is a tree-based, recursive partitioning machine learning approach. Within the forest, each tree depends on the values of a random vector sampled independently and with the same distribution for all other trees. The algorithm splits trees with the goal of reducing impurity between clusters of observations. Impurity is generally measured by information content metrics, such as the Gini score and residual sum of squares. Ultimately, the random forest gathers hundreds or thousands of trees to make predictions.

In predicting POS terminal adoption and share of P2B electronic payments at both the country- and merchant-level, we feed the random forest models with all the country- and merchant-level predictor variables, respectively (see S1 Appendix). This approach is common when data comes from surveys specifically designed to examine changes in banking and payment instruments [48, 49].

With the random forest estimation, our primary goal is to identify the variables that are most predictive of digital payment acceptance and usage. As explained further in the results section, the algorithm generates variable importance metrics for the model’s predictor variables. These model diagnostics provide inferential value, but they lack directionality and economic interpretation, as they are not in the units of response variables. Thus, perhaps the most important function of the random forest is that it helps us narrow down a core group of variables that are most predictive of digital payment acceptance and usage. We are able to use these predictors to more thoroughly explore the drivers of digital payment acceptance and usage by using the conditional inference tree and the causal forest.

### 3.4 Decision sequencing with conditional inference trees

After identifying predictors using random forest algorithms, we use the characteristics and determinants with the largest discriminant power to build a decision tree for each dimension by estimating a conditional inference tree [43]. This technique estimates a regression relationship through binary recursive partitioning in a conditional inference framework. In particular, the algorithm tests the global null hypothesis of independence between each of the input variables and the response and selects the input variable with the strongest association to the response. The algorithm then implements a binary split in the selected input variable and recursively repeats this process for each of the remaining variables. Importantly for our purposes, the conditional inference tree shows the sequential combination of factors that explain the EPA and usage decision process. This approach does not require any linearity assumptions, which is important because many of the digital payment acceptance and usage determinants could be related nonlinearly.

The conditional inference tree is similar in many ways to typical regression and classification trees, including those underlying random forests, in that it is a tree-based, recursive partitioning machine learning approach. A key difference, though, is that it is more statistical in nature, since it uses chi-square tests of independence to determine tree splits. As its name implies, the conditional inference tree is more geared toward “inference” than other tree-based methods. This technique provides insights into examining the tree structure in addition to focusing on predictive accuracy.

### 3.5 Estimating impact of incentives with causal forest estimation

Since machine learning models have not, historically, been designed to estimate causal effects, a new field of study has emerged over the last few years that combines the advances from machine learning with the theory of causal inference [50]. The aim of these techniques is to complement, rather than to serve as a substitute for, traditional machine learning methods, by helping researchers leverage the data-driven nature of machine learning to estimate causal effects [51, 52]. The main advantage of causal machine learning is that it can be used after the modeling phase in order to confirm some of the relations between predictors and the response variables.

In our context, by employing a causal learning method, we aim to examine the impact of incentives or conditions with the largest predictive power on the digitalization process. In a broad sense, it consists of comparing an outcome in a treated group (e.g., countries/merchants exposed to an incentive) with an untreated group (e.g., countries/merchants not exposed to an incentive). This departs to some extent from the sampling and treatment analysis in field experiments where sample selection and treatment implementation is under the control of the researchers, using randomization. In our setting, the treatment is associated with a quasi-natural experiment as we can combine a sample of merchants in different countries where some EPA incentives have been implemented as opposed to other countries or sub-samples of merchants with no access to such incentives. While the extension of our results to other jurisdictions not considered in our analysis should be done cautiously, we believe the causal forest analysis may help understand the impact of the incentives and conditions that impact EPA and usage controlling for a large number of covariates at both the country and the merchant level.

Among the recent methods developed in the causal machine learning literature, causal forest has gained particular relevance [44, 51, 52]. [53] shows that causal forests perform consistently well across different data generating processes and aggregation levels. The algorithm allows for a tractable asymptotic theory and valid statistical inference by extending the random forest algorithm. Methodologically, causal forests maintain the main structure of random forests, including recursive partitioning, subsampling, and random split selection. However, instead of averaging over the trees, causal forests allow for the estimation of heterogeneous treatment effects [45] by identifying how different treatments (e.g., incentive vs no incentive) affect the outcome (e.g., digital payment acceptance and usage). One important requirement for a proper identification is the so-called ‘honesty’ condition. This is the basic idea is that you cannot use the same outcome data to both partition the tree and estimate the average impact. This is particularly important when, rather than the standard randomization of samples, we use data to explore an exogenous change (i.e. policy) on a number of subsamples [52]. The ‘honesty’ condition is satisfied in our sample.

Compared to a normal decision tree, the causal tree uses a splitting rule that explicitly balances two objectives: (1) finding the splits where treatment effects differ the most; and (2) estimating the treatment effects most accurately. In order to obtain consistent estimates of the treatment effects (in our case, the features that may have an impact on digital payment acceptance and usage), the algorithm splits the training data into two subsamples: a splitting subsample and an estimating subsample [44, 53]. The splitting subsample is used to perform the splits and grow the tree, while the estimating subsample is used to make predictions. All observations in the estimating subsample are dropped down the previously grown tree until they fall into a terminal node. Ultimately, the prediction of the treatment effects is given by the difference in the average outcomes between the treated and the untreated observations of the estimating subsample in the terminal nodes. [45] provide a full mathematical explanation on how causal forests are built for causal inference.

Using this empirical methodology, we are able to examine the impact of those features with the largest predictive power on digital payment acceptance and usage. All analyses are carried out using the R package grf [54]. In running the algorithm, in the case of the country-level sample, we take a conservative approach by assuming that the level of digital payment acceptance and usage can be arbitrarily correlated within a country. Hence, the errors are clustered at the country-level.

## 4 Results

In this section, we discuss the results of the three empirical parts of our analysis—random forest, conditional inference trees, and causal forest.

### 4.1 Random forest results

Employing the random forest algorithm, we identify the best predictors for POS terminal adoption and MSMRs’ share of P2B digital payments. From over the 190 factors, we identify 14 variables with the largest predictive power of MSMRs adoption of POS terminals and consumer usage of digital payments at MSMRs. The random forest algorithm generates a variable importance ranking. The relative statistical importance of each factor in predicting the impact on the dependent variables is estimated. We measure the importance of each predictor by mean decrease in accuracy and mean decrease in Gini [55]. The mean decrease in accuracy reflects the mean loss in accuracy when each specific variable is excluded from the algorithm. Therefore, the determinants and characteristics with the greater mean decrease in accuracy are the most relevant. Additionally, the mean decrease in Gini is a measure of how each feature contributes to the homogeneity between the decision trees used in the resulting random forest.

This analysis provides eight variable importance plots from multiplying two accuracy methods (mean decrease in accuracy and mean decrease in Gini) by two dependent variables (POS terminal adoption and share of P2B electronic payments) covering two samples (country-level and merchant-level). For simplicity, Table 1 offers the factors with the largest prediction power for the two dependent variables that are consistently shown at the country- and merchant-level sample. The detailed variable importance plots are available upon request. In addition, Table 1 shows the predictors with mean decrease in accuracy larger than 10 percent and mean decrease Gini larger than 2 percent. Furthermore, our selection is consistent with the procedure proposed by [3]. It consists of 1) running the random forest algorithm and returns the mean decrease in accuracy and the mean decrease in Gini of each variable, 2) ranking every variable using the mean decrease in accuracy and the mean decrease in Gini, respectively, 3) scoring each variable, 4) computing the total score of each variable, 5) reordering them by the total score.

**Table 1:** Main predictors of POS adoption and share of P2B electronic payments (combined results from the country-level and merchant-level samples).

Response variable	Category	Predictor	Variable importance confirmed in:	
			Country-level sample	Merchant-level sample
POS	Merchant	Share of P2B electronic payments	✓	✓
Terminal	Payment	Merchants’ beliefs about consumer payment	n.a.	✓
Adoption	Chain	preferences		
		Percentage of wages paid electronically at the	n.a.	✓
		merchant level		
	Infrastructure	Information and Communication Technologies	✓	✓
		Account ownership	✓	✓
		National ID	✓	✓
	Institutional	Merchant fiscal incentives		✓
	and Policy	National financial inclusion strategy	✓	✓
		Wages paid into a transaction account	✓	✓
		Shadow Economy	✓	✓
Share of	Merchant	POS terminal adoption	✓	✓
P2B	Payment	Merchants’ beliefs about consumer payment	n.a.	✓
Electronic	Chain	preferences		
Payments		Percentage of wages paid electronically at the	n.a.	✓
		merchant level		
	Instruments	Previous card penetration	✓	✓
	Infrastructure	Information and Communication Technologies	✓	✓
	Institutional and	Wages paid into a transaction account	✓	✓
	Policy	Killer app		✓
	Access Points	POS adoption	✓	✓
		Agents of payment services providers	✓	n.a.
**Out-of-sample accuracy (70/30% split) of the random forest**				
POS adoption		89.91%		
Share of P2B electronic payments		92.14%		

**Note:** Predictors selected from the variable importance plots obtained from the random forest algorithm. The grouping of the variables follows [58].

“n.a.” notes that the variable was not available for that sample

For POS terminal adoption, the main predictors correspond to three variable groups: merchant payment chain, ICT infrastructure and account ownership, and institutional and policy actions. Merchant payment chain includes the MSMRs’ share of P2B digital payments, merchant perceptions on consumers payment instrument preferences, and the percentage of wages paid digitally at the merchant level. For actual usage of P2B digital payments at MSMRs, the merchant’s perception of consumer willingness to use payment cards, and merchant usage of digital payments to pay their workers are the main predictors of greater POS terminal adoption and greater share of digital payments. In addition, we might expect that over time, as the total MSMR’s share of P2B payments increases, more MSMRs would adopt POS terminals given the popularity of payment cards. However, in some countries, digital payments not requiring POS terminals have leapfrogged payment cards.

For infrastructure, our results suggest that ICT infrastructure, transaction account ownership, and national ID programs are strong predictors of POS terminal adoption. ICT infrastructure increases the ability to open transaction accounts especially remotely and access accounts to make digital payments. Because debit cards are linked to transaction accounts, account ownership is necessary for the adoption of debit cards by both consumers and MSMRs. Not surprisingly, national IDs are a strong predictor of POS terminal adoption because IDs enable widespread ownership of transaction accounts which are necessary for consumer card adoption which is a critical factor for merchants when deciding to install POS terminals.

We also find institutional and policy actions taken by policymakers and the size of the shadow economy are important predictors of POS terminal adoption. Our empirical results regarding the impact of the shadow economy are robust to the use of some alternative measures of economic informality. In particular, our results remain very similar when we use alternative indicators of informality as the informality measures based on dynamic general equilibrium models and on the combination of multiple indicators, as provided by [56]. Correlation across the 106 countries in our sample between our shadow economy metric and these economic informality indicators ranges from 87 percent to 89 percent. Public authorities have implemented financial inclusion programs to increase transaction account ownership often with access to debit cards. Fiscal incentives for merchants are also strong predictors for greater POS terminal adoption. Furthermore, payment of wages into transaction accounts is also a strong predictor of greater adoption of POS terminals by MSMRs. Finally, the size of the shadow economy is also a strong predictor MSMR POS terminal adoption. The larger the size of the shadow economy the lower the likelihood of POS terminal adoption.

We also identify strong predictors of the MSMRs’ share of P2B electronic payments of which four of them are the same for POS terminal adoption. We categorize these predictors into four groups: merchant payment chain, payment instrument developments, ICT infrastructure and account ownership, policy variables, and access points. In the MSMR payment chain category, three variables are strong predictors of the MSMRs’ share of P2B electronic payments: MSMRs’ beliefs about consumer payment preferences, the percentage of the total value of electronic wage payments, and the proportion of the electronic payments made to suppliers. As discussed before, as MSMRs become more digitally liquid, they will tend to adopt digital payments for all incoming and outgoing payments. Given that cards are still a popular digital payment, POS terminal adoption continues to be a strong predictor of MSMRs’ share of P2B digital payments.

In the payment instruments group, previous (debit and credit) card penetration (measured as the penetration in 2011) are strong predictors of greater the share of MSMRs’ P2B electronic payments. This result suggests that consumers may require time to change their payment habits and merchants will install POS terminals as consumer demand increases over time. There may also be spillover effects between some merchant sectors into others. For example, in many countries, high-end merchants and merchants located in tourist locations are likely to be early adopters of payment cards. As consumer and merchant awareness of digital payments increases, the adoption of POS terminals by other types of MSMRs may also increase.

As expected, the level of development of ICT infrastructure is a strong predictor for the MSMRs’ share of P2B digital payments. This result is more general than the result with POS terminal adoption because usage of noncard digital payment instruments is included. In the future, we would expect that payment cards will continue to face greater competitive pressure from alternative payments such as those based on fast payment networks and closed-loop digital payment networks.

In the policy and access points categories, wages being paid in a transaction account, presence of a killer app, POS terminal adoption, and presence of agents for payment service providers are strong predictors for MSMR P2B digital payments. This result suggests that there are adoption and usage synergies between digital payments across the MSMRs’ payment chain even if the payment instruments themselves may differ. In addition, workers that are receiving payments digitally may prefer to pay digitally at MSMRs if given the opportunity.

One key finding is that each of the dependent variables is a predictor for the other dependent variable. In the case of MSMRs’ share of electronic P2B payments, POS terminal adoption provides a means to accept payment cards, the most popular digital payment option at MSMRs during our sample period. For predicting POS terminal adoption, the MSMRs’ share of P2B electronic payments is a strong predictor. The result can be interpreted as a confirmation of the feedback mechanism, whereas POS adoption increases, more MSMRs adopt POS terminals. In other words, as more MSMRs install POS terminals, other MSMRs also adopt because if they do not, they may lose business to MSMRs that accept digital payments. However, as new digital payment instruments that do not rely on POS terminals have greater market penetration, we would expect this effect to lessen.

### 4.2 Conditional inference tree results

While the random forest estimation techniques identified predictors in isolation, conditional inference trees allow us to study how a combination of factors and their sequence increase the likelihood of POS terminal adoption and MSMRs’ digital share of P2B payments. Based on the predictors from the variance importance analysis, we estimate conditional inference trees that identify the interactions among predictors and their sequences.

**Fig 1:**
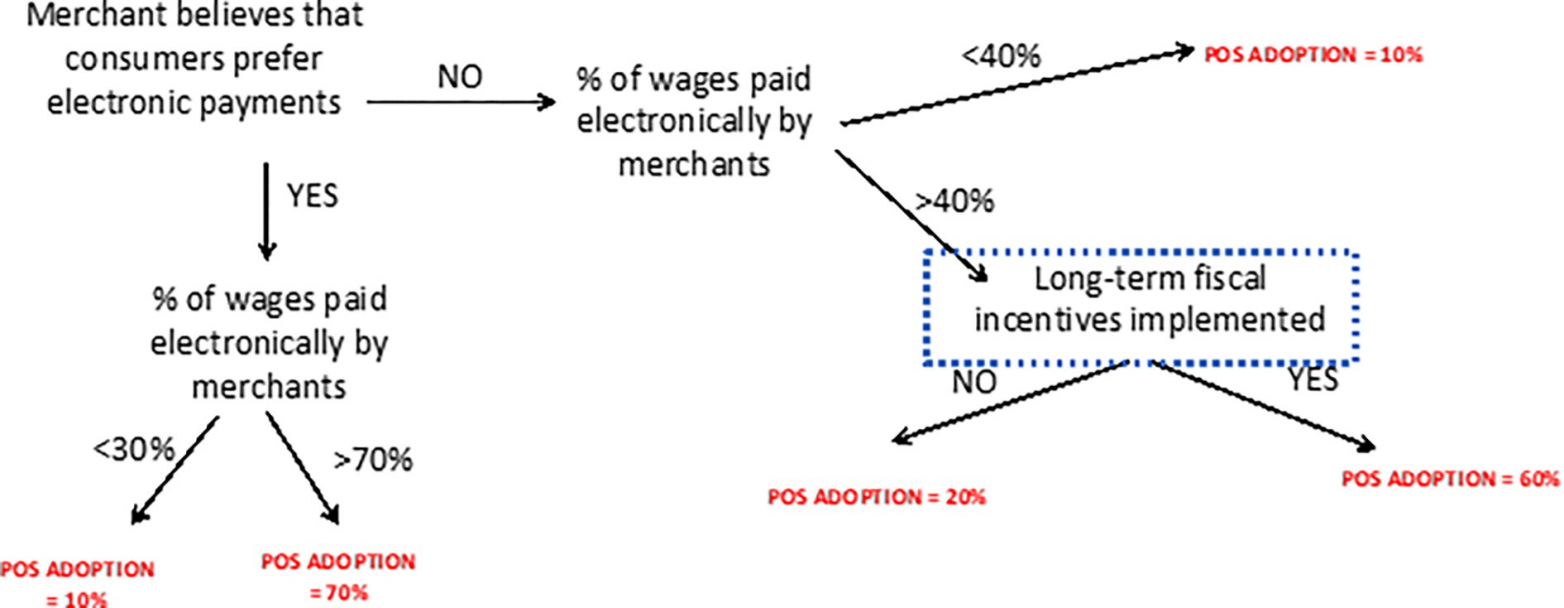
Example of a conditional classification tree. **Note:** This conditional inference tree is obtained from a merchant-sample estimation using the parameters provided by the variable importance plot from the random forest.

An example of a conditional inference tree is shown in Fig 1. In this example, we observe that if MSMRs believe that consumers prefer electronic payments and percent of wages paid electronically is greater than 70 percent, the likelihood of MSMR POS terminal adoption is 70 percent. Alternatively, if MSMRs believe that consumers do not prefer digital payments and less than 40 percent of wages are paid electronically by MSMRs, the likelihood of MSMR POS terminal adoption is only 10 percent. If consumers do not prefer electronic payments, the percentage of wages paid digitally is greater than 40 percent and if long-term fiscal incentives are implemented, the likelihood of MSMR POS adoption is 60 percent suggesting the importance of fiscal incentives especially in the absence of merchant beliefs that consumers do not prefer digital payments.

For simplicity, we offer a summary of the most impactful prediction relationships in Tables 2 and 3. These estimates are derived from numerous conditional classification tree analysis similar to Fig 1 that are not shown but available upon request. In Table 2, we summarize the conditional paths that have the most impact on MSMR POS terminal adoption. Interestingly, POS terminal adoption is 200 percent more likely when the MSMRs’ share of electronic P2B payments and bank account ownership are above the median country (23.2 percent and 62 percent, respectively). These thresholds may be challenging for a number of countries within the sample. The 25th percentile for share of electronic P2B payments and bank account ownership are 9.2 percent and 44.2 percent respectively. The median percentage of electronic P2B transactions at MSMRs is a relatively low threshold. This result reinforces the dominant role of payment cards among the digital payment choices generally available.

**Table 2:** Factors influencing POS terminal adoption.

Factors for POS Terminal Adoption	Increase in Likelihood of POS Adoption
Share of P2B electronic payments and bank account ownership is above the median country (country level)	200 percent
Merchants believe consumers prefer electronic payments and wages are paid electronically (mid-high or high preference) (merchant level)	100 percent
Wages are paid electronically and combined with account ownership or with ICT infrastructures above median (country level)	100 percent
ICT or national ID implementation are over the median value and shadow economy below 15% (country level)	50 percent
Shadow economy below 25% is combined with implementing a national financial inclusion strategy or with merchant fiscal incentives (country level)	20 percent

**Table 3:** Factors influencing share of electronic P2B payments.

Factors for P2B Electronic Transactions	Increase in Likelihood of P2B Electronic Share
Wages paid electronically and card penetration in previous 5 years are over median value (country level)	100 percent
POS terminal adoption is mid high or high (country level)	60 percent
ICT above median is combined with killer apps or with a significant use of agents of payment services providers (country level)	50–60 percent
Merchants believe consumers prefer electronic payments and electronic payments to suppliers above median value (merchant level)	30 percent

We also find that if wages are paid electronically above the median (35 percent) and merchants believe that consumers prefer electronic payments is also above the median (52 percent), POS terminal adoption is 100 percent more likely. In addition, there may be a feedback loop whereby as more consumers prefer digital payments, more merchants install POS terminals leading to more consumers adopting and so forth. As consumers become more accustomed to using payment cards, the demand for merchants that did not previously adopt payment infrastructure increases. In addition, payment card acceptance may be a strategic tool to steal customers from merchants that do not accept cards. Unfortunately, our data does not allow us to study business stealing. For more on business stealing in a theoretical context, see [57]. Consumer preferences toward using digital payments have likely improved because of their access to transaction accounts and payment cards.

Also, if at least 35 percent of wages are paid by a given MSMR into a transaction account at a bank (the median in our sample) and ICT infrastructure is higher than the median country (above 5.3 on a 10-point scale). The 25^th^ percentile values for wages paid in a financial institution account and the ICT index are 10 percent and 3.5, respectively. POS terminal adoption is twice as likely. This result suggests that if merchants pay a sufficient number of workers digitally into a bank account (35% of more), they are more likely to accept card payments from their customers if there is a sufficient level of ICT infrastructure. Our results suggest that public policy should not only focus on P2B but also consider incentives to increase B2P payments such as wages.

We also find that with ICT infrastructure above 5.3 on a 10-point scale or national ID implementation being above median (94 percent) and the proportion of the shadow economy to the whole economy being no greater than 15 percent result in a 50 percent greater likelihood of MSMR POS terminal adoption. As we discussed above, ICT infrastructure is important for POS terminal adoption, but other factors may be necessary. In this case, a national ID system enables greater ownership of transaction accounts debit card.

Furthermore, implementing a national financial inclusion strategy or merchant fiscal incentive initiative (at a national level) and having a proportion of the shadow economy to the whole economy below 20 percent results in the likelihood of POS terminal adoption increasing by 20 percent. The implementation of a financial inclusion strategy is another variable that captures a necessary condition for card ownership which in turn increases the likelihood of MSMR POS terminal adoption. The merchant fiscal incentive reduces the benefits of tax evasion.

In Table 3, we report our results on the factors that impact the MSMRs’ share of electronic P2B payments. When wages are paid digitally and card penetration in the previous five years are above the median (35 percent and 11 percent, respectively), the likelihood of P2B electronic payments increases by 100 percent. This result suggests that greater awareness by consumers in the form of greater access to payment cards or by greater payment of their wages digitally, increases the likelihood of digital payments increases substantially.

Given the popularity of cards as a digital alternative to cash, if POS terminal adoption is over the median (10,005 terminals per 100,000 adults) the predicted share of P2B digital payments increases by 60 percent. Alternatively, it is less likely that merchants will adopt POS terminals if they believe that consumers do not have access to them or will not use them.

If the level of ICT is over the sample median (5.4 out of 10) is combined with the development of killer apps, or with a significant use of agents of payment service providers (1.2 per 1,000 inhabitants), P2B electronic payments’ likelihood increases by 50–60 percent. Killer apps allow payments to be made by mobile phones and may serve as an alternative to payment cards. However, sufficient ICT infrastructure is likely necessary along with innovative mobile phone-based solutions to increase MSMR’s P2B digital share of payments. In the treatment effects section, our results suggest a negative relationship between leapfrogging and POS terminal adoption. However, card payments could also benefit from mobile phone technology and QR codes but this likely occurs when a robust card ecosystem already exists. Also, the importance of agents suggests that digital payments do not immediately lead to digital liquidity, but cash agents are generally required for consumers and businesses to convert digital funds to cash and vice versa. We would expect a greater reliance on cash agents when significant parts of the population are unbanked or do not use banks, or a lack of merchant acceptance of digital payments.

When payments to direct suppliers of MSMRs are above the median sample value (45.9 percent) and the wages are paid electronically (over the median value), the MSMRs’ share of P2B electronic payments is predicted to increase by 30 percent. This result suggests that digital payments in other parts of the merchant’s payment chain likely increases usage of digital P2B payments.

### 4.3 Causal forest results

While the random forest and conditional inference trees offer insights into strong predictors of digital payment acceptance and usage along with their sequencing and interactions with each other, we also investigate causal effects. Specifically, we estimate the impact of a change in each explanatory variable on one of our two dependent variables. The treatment effect is given by the difference in the average outcomes between the treated and the untreated observations. The standard procedure is to compute the “average treatment effects” (ATE) when the treatment is defined based on a binary outcome as is the case for some incentives in our database. For example, if we compute the impact of financial inclusion policies on POS terminal adoption, we can estimate how POS adoption is impacted on average in countries with these policies compared with countries where no such policies exist, controlling for the rest of explanatory factors. Our analysis is not based on a randomized experiment but, instead, our analysis is a natural experiment based on exogeneous EPA incentive policies in various countries for different types of merchants.

We need to consider that our data also have a number of continuous variables. In this case, instead of the ATE, we compute the “average partial effect” (APE), which shows the percent change in the dependent variable due to a unit change in the treatment variables. Given the four-level (1 = low; 2 = mid-low; 3 = mid-high; and 4 = high) transformation of the continuous explanatory variables in our setting, the APE will show the average change in the dependent variable of going from one level of the treatment to the next one. For example, if we select the “ICT development index” as a treatment for POS adoption, we will be showing the average partial effect on POS adoption of a country moving from one level to a higher one (e.g., from mid-low to high ICT group).

As an initial test, we consider the top thirty explanatory factors in the variable importance plot as a reference. We find significant relationships for 23 variables. We group these variables into six categories: access points, economic formality, ICT infrastructure and account ownership, policy variables, instruments, and merchant payment chain. We run a causal forest for each one of the 23 variables and replicate the process for both dependent variables in the country-level and the merchant-level samples. All the results are shown as point estimates with 95 percent level confidence intervals.

**Fig 2:**
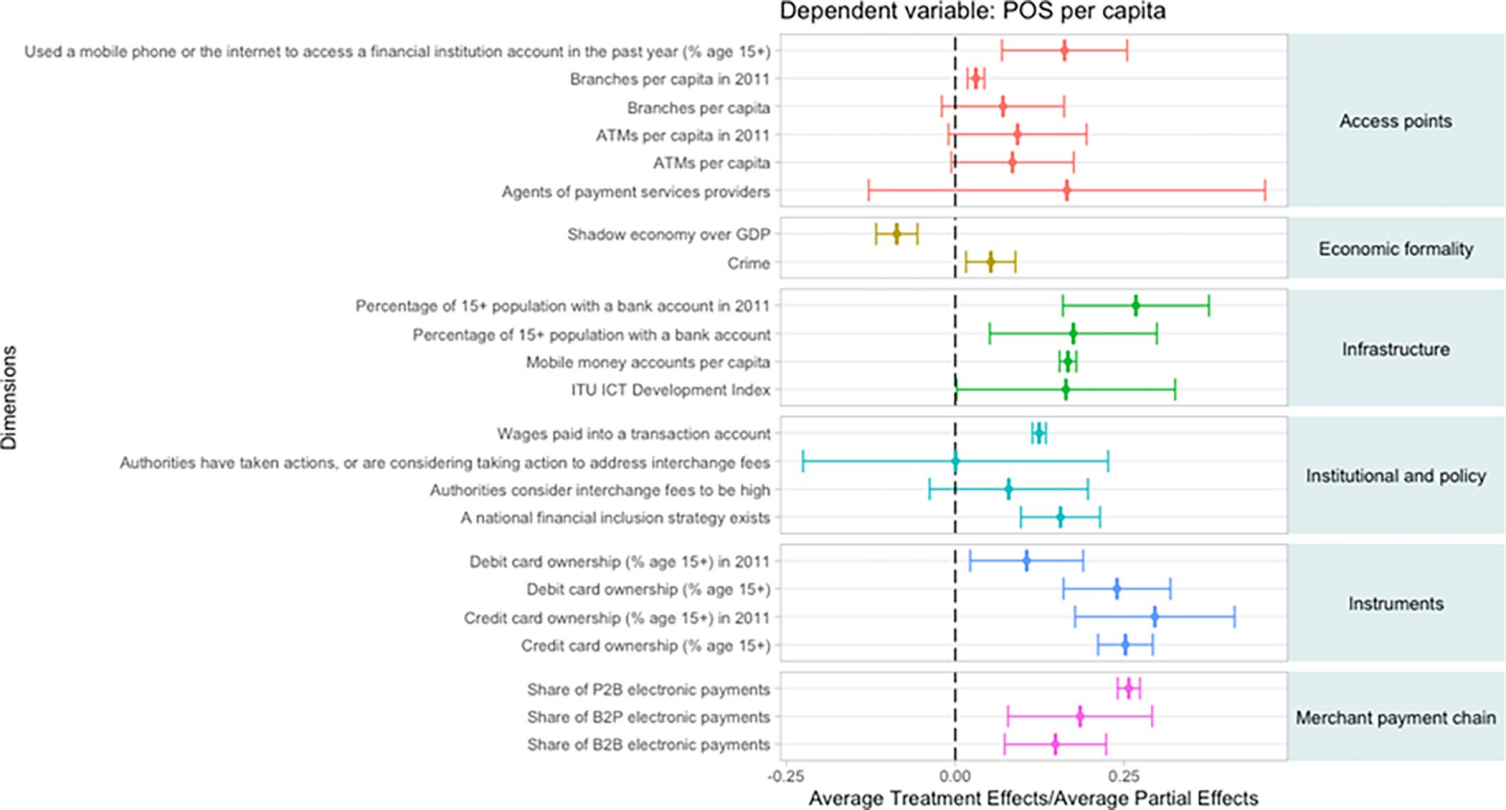
Treatment effects on POS adoption (country-level sample).

In Fig 2, we report the results for the country-level sample where POS terminal adoption is the dependent variable. In the case of access points, the use of “mobile phone or the internet to access a financial institution account” is found to have a significant impact (16.2 percent) on average across the four levels of POS terminal adoption and treated and untreated countries, suggesting that consumers’ financial digitalization significantly influences merchants’ adoption of POS terminals. Among more traditional channels, bank branches per capita in 2011 seem to have a more limited positive effect.

MSMRs may have a greater incentive to accept cash as a means to evade taxes. On the other hand, greater reliance on cash transactions may result in MSMRs and consumers. In the case of economic formality, the treatment effect of the “shadow economy over GDP” on POS adoption is, as expected, negative with a relatively tight confidence interval illustrating that a large shadow economy reduces the incentive for MSMRs to adopt POS terminals on average. The effect of “crime” (measured as robbery and assaults per inhabitant) is positive with very tight confidence interval suggesting that safety concerns for consumers and MSMRs leads to greater adoption of digital payment acceptance infrastructure.

Account ownership indicators are among those with the larger marginal impact on POS terminal adoption, suggesting that financial inclusion policies are impactful and should be encouraged. We capture long-term transaction account ownership at banks by considering 2011 data. This is a proxy for a time dimension. Changing consumers’ and MSMRs’ payment behavior requires time and these variables are able to provide some insights. For short-term bank transaction account ownership, we use 2014. Interestingly, we find that the impact for long-term account ownership is higher than short-term account ownership suggesting that consumers need time to change from their preference for cash transactions at POS. As for technology-driven changes, while there is a positive APE on POS terminal adoption of mobile money accounts per capita possibly suggesting that similar infrastructures are used for noncard and card payments.

Two policy actions are economically significant and positive. These include mandates to pay wages into transaction accounts and the presence of a national financial inclusion strategy. The marginal impacts of both these public policy initiatives are significant.

From a price regulation perspective, we tried to capture the impact of regulating interchange fees, the fees that the merchant’s bank pays the cardholder’s bank and comprises the bulk of the merchant cost to accept payment cards. The two variables that we consider are: “authorities have taken actions or are considering taking action to address interchange fees” and “authorities consider interchange fees to be high.” We would expect both of these variables to have negative impact on POS terminal adoption because high fees would deter merchant adoption of POS terminals. However, the treatment effects of these variables do not have the expected sign and are insignificant. This result suggests that interchange fees may not be the main deterrent for the lack of POS terminal adoption. However, we caution that these results are averages across countries in different stages of financial development. In some cases, interchange fee regulation occurs in countries where merchant adoption is near complete. In these cases, interchange fee regulation is not likely to increase adoption by merchants or usage by consumers. In some cases, interchange fee regulation was implemented to reduce certain types of payment card transactions. In other cases, interchange fee regulation occurs in countries where the adoption and usage of cards by consumers is very low. Decreasing interchange fees may not provide incentives to consumers to adopt and use payment cards. Alternatively, the lack of a payment card infrastructure may result in new payment technologies being adopted such as noncard based mobile payments. This result is also consistent with the premise that there are other factors besides payment card fees that determine whether consumers will use payment cards even if merchants accept them.

Our next category is payment instruments. While debit and credit card ownership have a significant impact on POS terminal adoption, the latter seems to have a larger effect and to increase over time. The link between payment cards and POS terminals should not be surprising since payment card acceptance for the most part requires a terminal. Alternative acceptance infrastructure has recently been introduced but the card form factor remains the dominant one. In many countries, credit cards are accepted at tourist locations and certain high-end stores well before adoption of debit cards by the masses. Consumer awareness and card infrastructure grows from credit card acceptance in certain sectors which in turn may allow for broader acceptance of debit cards.

As we have seen before, digital payment usage in other parts of the MSMR’s payment chain positively impacts POS terminal adoption. The impact of the share of P2B, the share of B2B and the share of B2P electronic payments over total transactions on POS terminal adoption is positive and significant. The P2B results suggest a reinforcing feedback loop.

**Fig 3:**
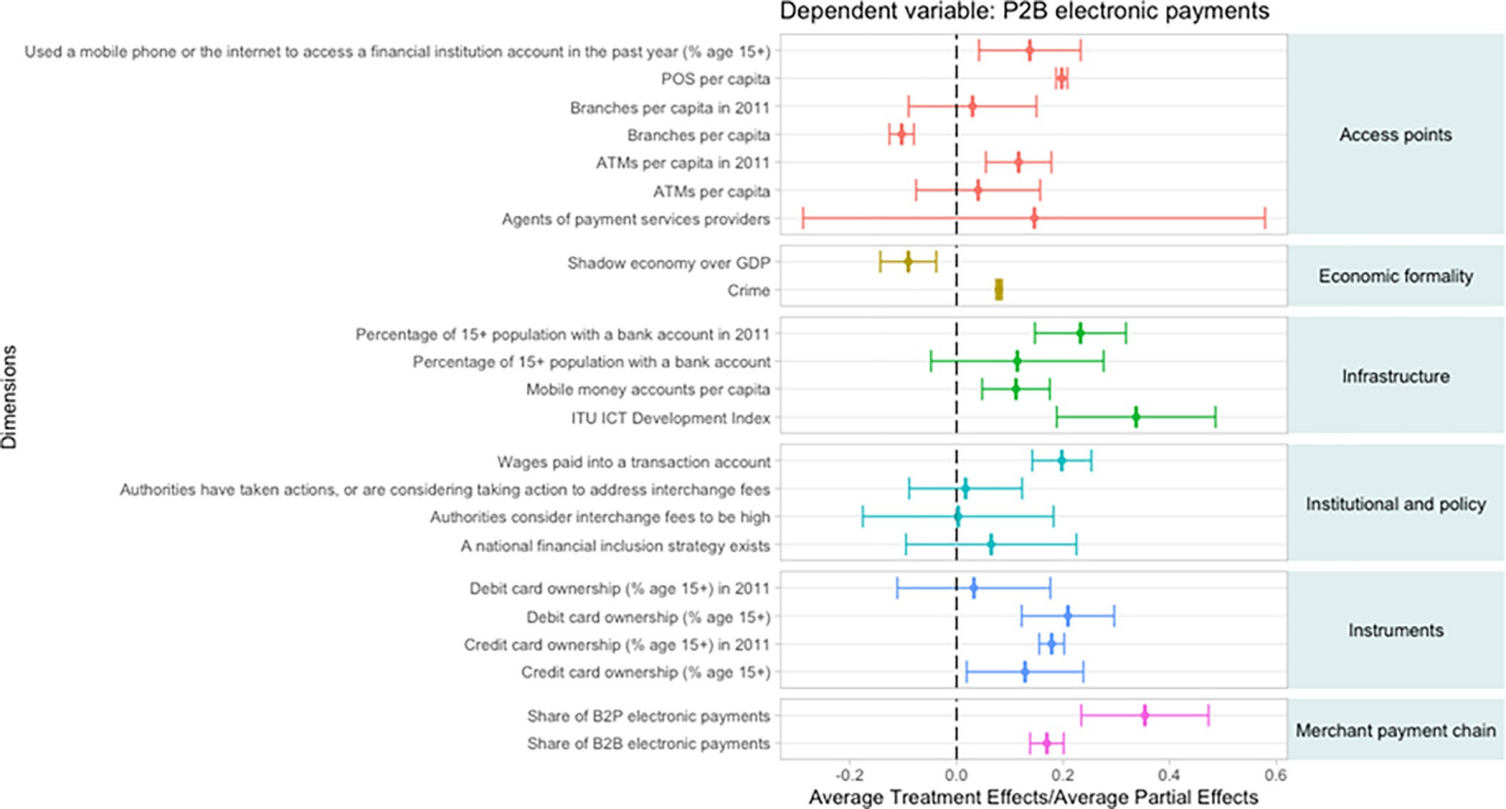
Treatment effects on the share of P2B electronic payments (country-level sample).

The impact on the MSMRs’ share of P2B electronic payments at MSMRs for the country-sample are shown in Fig 3. For access points, not surprisingly, using the internet or mobile phone to access a transaction account, POS terminal per capita, and ATMs per capita in 2011 are significant and positively impact MSMR share of digital P2B payments. However, branches per capita is found to have a negative impact, suggesting the persistence of cash usage has a significant effect in countries with large bank physical networks also the lower shoe leather costs may help maintain the demand for cash transactions.

For the infrastructure variables, there is one main difference between the POS terminal adoption and MSMR digital share of P2B payments. The effect of ICT infrastructure for the latter is large and positive suggesting that all digital payments require sufficient ICT infrastructure.

For government policy actions, the national financial inclusion strategy variable does not remain significant suggesting that other factors such as technological advancements can reduce the importance of a national financial inclusion strategy. While we discuss the impact of a considerable number of incentives, others (such as terminal subsidies) or lotteries could not be empirically analyzed due to data availability. In addition, the percentage of population with a bank account is insignificant suggesting that bank accounts may not be a precursor in all countries for greater digital payment usage.

In the case of payment instruments, debit card ownership in 2011 does not remain significant while the other variables remain positive and significant. Given new data that captures noncard based payments, we would expect the importance for card adoption to decrease even more. Card networks are also processing other types of payments such as fast payments using their infrastructure.

The magnitudes for the payment chain variables increase suggesting that merchants may adopt other digital payments besides cards. As for the merchant payment chain, P2B payments seem also to be substantially driven by B2B payments and, in particular, by B2P payments.

The merchant-level sample allows us to compare treated and untreated merchants for each incentive. Because our incentives occur at the national level, our comparison will be between merchants receiving the incentive in one or more countries (treated group) against merchants not receiving the incentive in other countries (untreated group).

**Fig 4:**
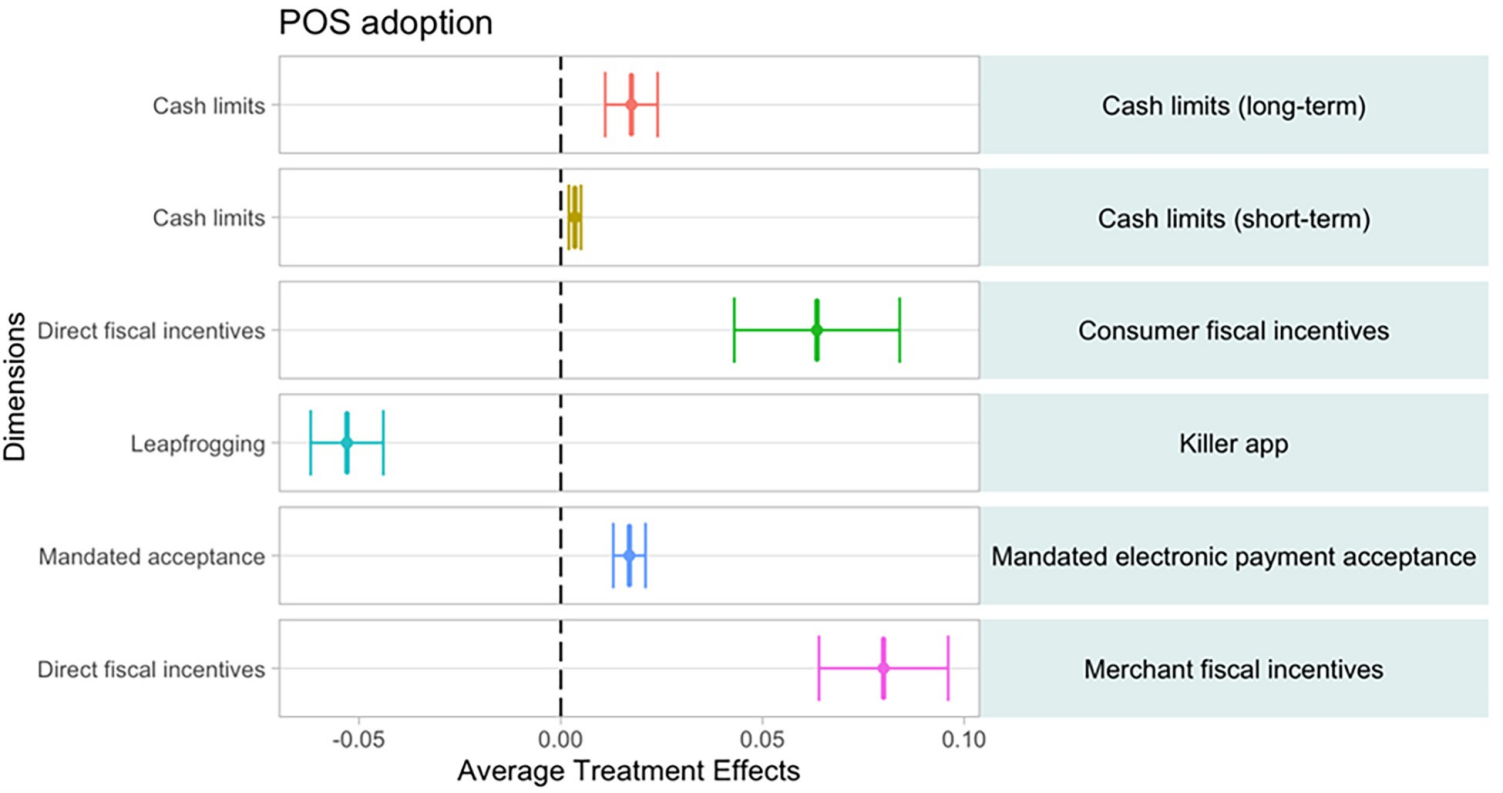
Impact of incentives: Treatment effects on pos terminal adoption (merchant level sample).

For expositional simplicity, we only report the impact of those incentives not previously included in the country-level sample (Fig 4). Implementing merchant fiscal incentives will increase the likelihood of POS terminal adoption. Consumer fiscal incentives also have a positive and significant effect. The impact is lower for mandated acceptance and for cash limits. We also find that in those cases where a leapfrogging strategy develops, e.g. a killer mobile app for payments, the effect is negative because POS terminal adoption may no longer be necessary. The adoption of dongles on mobile phones and quick response codes may eventually eliminate the need for terminal adoption.

**Fig 5:**
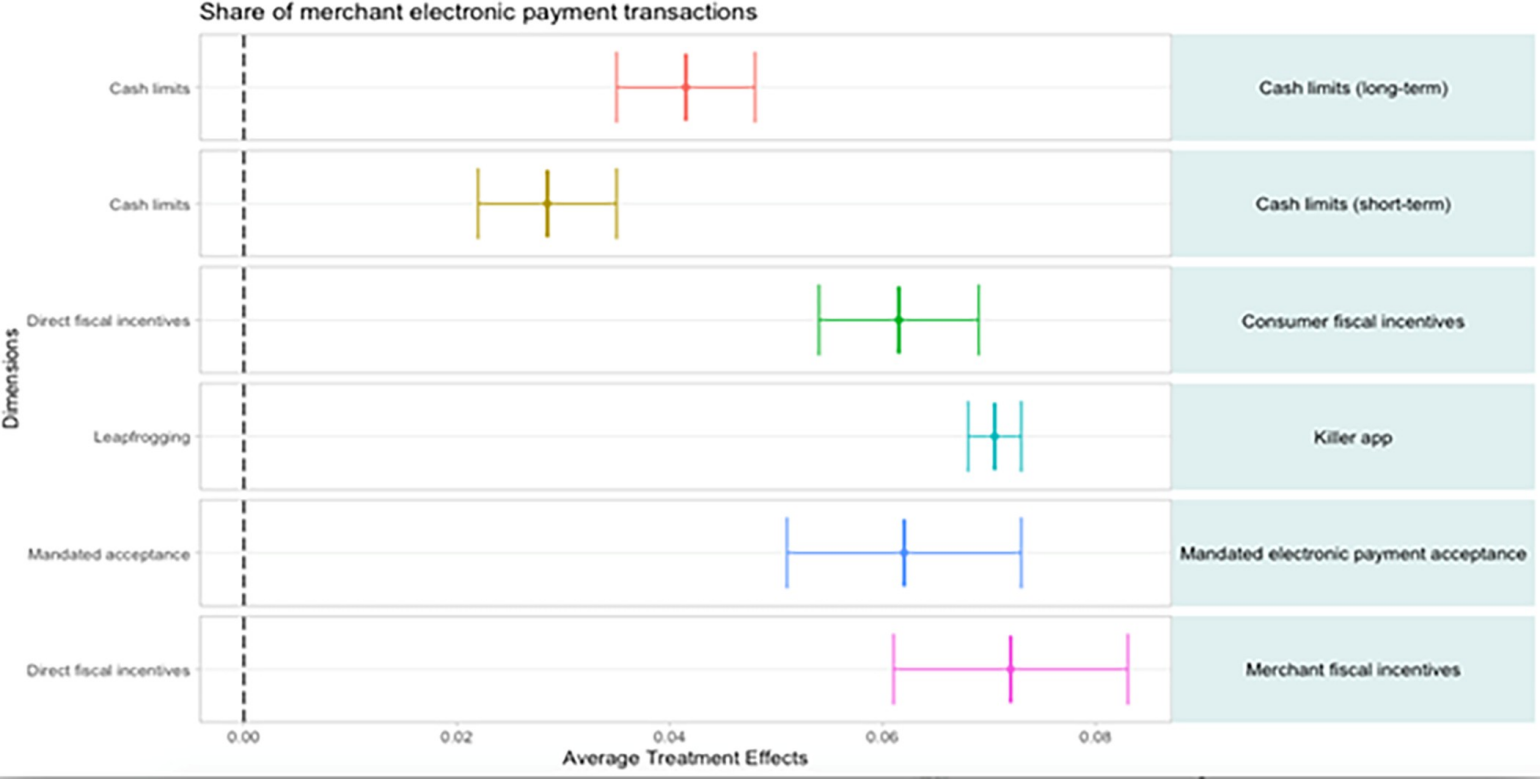
Impact of incentives: Treatment effects on the share of merchant P2B electronic payment transactions (merchant-level sample).

Fig 5 reports the merchant-level effects of the different incentives on the share of P2B electronic payments. In this case, the effect of the killer app is positive and the largest among the incentives considered showing the effects of mobile-related technology adoption on payment usage. Merchant and consumer fiscal incentives are also found to have significant effects and the effect is also considerable in the case of mandated use of electronic payments. The impact of short-term and long-term cash limits are also positive but not as large.

A summary of all treatment effects for the country-level sample and the merchant-level sample is shown in Tables 4 and 5, respectively. Overall, the empirical analysis provides some generalized conclusions to increase merchant acceptance and usage of digital payments. First, merchant and consumer decisions are interlinked when it comes to adoption and usage of digital payments. Second, digital payment acceptance and usage are positively impacted when any part of the MSMR’s payment chain uses digital payments. Third, in cases where well-functioning payment infrastructure does not exist or is not widely used, certain technologies help MSMRs bypass traditional acceptance infrastructure and adopt digital payments via alternative routes (e.g., leapfrogging via mobile payments). Fourth, while several strategies and incentives can be effective to increase digital payment acceptance and usage, their impact depends critically on the formality of the economy.

**Table 4:** Summary of treatment effects from the country-level sample.

POS adoption						Share of electronic P2B payments			
variable	level	ATE/ APE	Lower bound	Upper bound	variable	level	ATE/ APE	Lower bound	Upper bound
Merchant payment chain	Share of P2B electronic payments	0.257	0.24	0.273	Merchant payment chain	Share of B2B electronic payments	0.170	0.138	0.201
Merchant payment chain	Share of B2B electronic payments	0.148	0.073	0.223	Merchant payment chain	Share of B2P electronic payments	0.354	0.234	0.473
Merchant payment chain	Share of B2P electronic payments	0.185	0.078	0.291	Institutional and policy	Wages paid into a transaction account	0.198	0.142	0.253
Institutional and policy	Wages paid into a transaction account	0.124	0.114	0.134	Institutional and policy	A national financial inclusion strategy exists	0.065	-0.095	0.225
Institutional and policy	A national financial inclusion strategy exists	0.156	0.097	0.214	Institutional and policy	Authorities consider interchange fees to be high	0.003	-0.176	0.182
Institutional and policy	Authorities consider interchange fees to be high	0.079	-0.038	0.196	Institutional and policy	Authorities have taken actions, or are considering taking action to address interchange fees	0.017	-0.089	0.123
Institutional and policy	Authorities have taken actions, or are considering taking action to address interchange fees	0.001	-0.225	0.226	Access points	Used a mobile phone or the internet to access a financial institution account in the past year (% age 15+)	0.138	0.042	0.233
Access points	Agents of payment services providers	0.165	-0.128	0.458	Access points	Agents of payment services providers	0.146	-0.288	0.579
Access points	Branches per capita	0.071	-0.02	0.161	Access points	POS per capita	0.198	0.187	0.208
Access points	ATMs per capita in 2011	0.092	-0.01	0.194	Access points	ATMs per capita	0.041	-0.076	0.157
Access points	ATMs per capita	0.085	-0.006	0.175	Access points	Branches per capita	-0.103	-0.126	-0.08
Access points	Branches per capita in 2011	0.031	0.043	0.018	Access points	ATMs per capita in 2011	0.117	0.055	0.178
Access points	Used a mobile phone or the internet to access a financial institution account in the past year (% age 15+)	0.162	0.069	0.254	Access points	Branches per capita in 2011	0.030	-0.09	0.15
Infrastructure	Mobile money accounts per capita	0.167	0.154	0.179	Infrastructure	Mobile money accounts per capita	0.112	0.048	0.175
Infrastructure	ITU ICT	0.164	0.002	0.325	Infrastructure	ITU ICT	0.337	0.188	0.486
Infrastructure	Percentage of 15+ population with a bank account	0.175	0.051	0.298	Infrastructure	Percentage of 15+ population with a bank account	0.114	-0.048	0.276
Infrastructure	Percentage of 15+ population with a bank account in 2011	0.267	0.159	0.375	Infrastructure	Percentage of 15+ population with a bank account in 2011	0.233	0.147	0.318
Instruments	Credit card ownership (% age 15+)	0.252	0.211	0.292	Instruments	Credit card ownership (% age 15+)	0.129	0.019	0.238
Instruments	Debit card ownership (% age 15+)	0.239	0.16	0.318	Instruments	Debit card ownership (% age 15+)	0.209	0.122	0.296
Instruments	Credit card ownership (% age 15+) in 2011	0.295	0.177	0.413	Instruments	Credit card ownership (% age 15+) in 2011	0.179	0.155	0.202
Instruments	Debit card ownership (% age 15+) in 2011	0.106	0.022	0.189	Instruments	Debit card ownership (% age 15+) in 2011	0.033	-0.111	0.176
Economic formality	Shadow economy over GDP	-0.087	-0.117	-0.056	Economic formality	Shadow economy over GDP	-0.091	-0.143	-0.038
Economic formality	Crime	0.053	0.016	0.089	Economic formality	Crime	0.080	0.074	0.085

Note: the table shows the average treatment effect (ATE) for binary variables and the average partial effect (APE) for four-level (1, 2, 3, 4) variables, along with the lower and upper bound of the confidence intervals.

**Table 5:** Summary of treatment effects from the merchant-level sample.

POS adoption					Share of P2B electronic payment value					Share of P2B electronic payment transactions				
variable	level	ATE	Lower bound	Upper bound	variable	level	ATE	Lower bound	Upper bound	variable	level	ATE	Lower bound	Upper bound
Merchant fiscal incentives	Direct fiscal incentives	0.080	0.064	0.096	Merchant fiscal incentives	Direct fiscal incentives	0.050	0.032	0.068	Merchant fiscal incentives	Direct fiscal incentives	0.072	0.061	0.083
Consumer fiscal incentives	Direct fiscal incentives	0.064	0.043	0.084	Consumer fiscal incentives	Direct fiscal incentives	0.042	0.029	0.054	Consumer fiscal incentives	Direct fiscal incentives	0.062	0.054	0.069
Mandated electronic payment acceptance	Mandated acceptance	0.017	0.013	0.021	Mandated electronic payment acceptance	Mandated acceptance	0.047	0.028	0.066	Mandated electronic payment acceptance	Mandated acceptance	0.062	0.051	0.073
Cash limits (long-term)	Cash limits	0.018	0.011	0.024	Cash limits (long-term)	Cash limits	0.062	0.041	0.082	Cash limits (long-term)	Cash limits	0.042	0.035	0.048
Cash limits (short-term)	Cash limits	0.004	0.002	0.005	Cash limits (short-term)	Cash limits	0.046	0.022	0.07	Cash limits (short-term)	Cash limits	0.029	0.022	0.035
Killer app	Leapfrogging	-0.053	-0.062	-0.044	Killer app	Leapfrogging	0.061	0.050	0.071	Killer app	Leapfrogging	0.071	0.068	0.073

Note: the table shows the average treatment effect (ATE), along with the lower and upper bound of the confidence intervals.

All the estimated treatment effects at the country-level and merchant-level are shown in Tables 4 and 5, respectively. We have also conducted a number of robustness tests to check the accuracy and stability of our results compared to other methods and model specifications. These are shown in S2 Appendix for exposition simplicity.

## 5 Policy implications

In this section, we discuss different government policies that would encourage greater acceptance and usage of digital payments. Merchant adoption of acceptance infrastructure does not necessarily translate into usage of payment cards. Interestingly, we find that newer technologies using the mobile phone channel increases adoption and usage of digital payments especially in countries where card penetration is low. In addition, we are able to identify which public sector incentives along with private sector enhancements such as killer apps result in greater usage of digital payments. Furthermore, our results suggest that a set of actions may be necessary by the public and private sectors to encourage the acceptance and usage of digital payments. We find the following government strategies may be successful to increase digital payment acceptance and usage:

1. Our analysis suggests that transaction account ownership whether at a bank or not generally increases merchant adoption of POS terminals and increases the share of MSMRs’ digital payments received from their customers. Countries should implement policies to promote greater financial inclusion.
2. Our analysis suggests that improvements in ICT infrastructures are likely to increase acceptance and usage in countries with high transaction account ownership. Countries should continue to invest in ICT infrastructure to enable greater digital payment acceptance and usage.
3. Our analysis suggests that when other parts of their payment chain are using digital payments, MSMRs are likely to increase their adoption of POS terminals resulting in more digital payments. Governments should encourage merchants to pay their workers electronically along with mandating digital payments for government wages.
4. Our analysis suggests that leveraging mobile phones to deliver payment services via killer apps may enable leapfrogging of card acceptance infrastructure using less expensive QR technology. Governments should encourage greater adoption of such technologies while maintaining adequate consumer protections and fraud prevention protocols.
5. Our analysis suggests that consumer and merchant fiscal incentives can be effective in increasing merchant acceptance and usage of digital payments. Reducing taxes paid by merchants and consumers are some of the most effective incentives to increase digital payment acceptance and usage. Governments should consider using fiscal incentives to encourage greater digital payment usage.
6. Our analysis suggests that cash limits and mandated digital payment acceptance are likely to increase digital payment acceptance and usage. Our analysis suggests that government policies targeted at reducing the shadow economy such as mandated use of electronic payments or transaction limits for cash transactions can be effective tools to increase EPA and usage although enforcement of these policies may be challenging.

## 6 Conclusion

In this paper, we provide an alternative approach to study how to identify key predictors to increase adoption and usage of digital payments by consumers and merchants. We consider hundreds of predictors and identify certain factors that increase the likelihood of adoption and usage. Finally, we are able to quantify the impact of certain incentives. Our results suggest that government initiatives such as increasing access to transaction accounts at banks and nonbanks, encouraging digital wage payments, and improvements to ICT technologies increase the acceptance and usage of P2B digital payments at MSMRs. Furthermore, the presence of killer apps that reside on mobile phones along with greater awareness of the benefits of digital payments increases P2B digital payments. Finally, our results suggest that policies targeted at reducing the size of the shadow economy, such as consumer and merchant fiscal incentives, cash thresholds on transactions, and mandated electronic payment acceptance, can be effective policies but often the least studied.

## 7 Limitations

Our study aims at providing new insights on the factors that influence the acceptance of digital payments internationally. However, machine learning weights prediction over inference and it is subject to interpretation. For these reasons, our results should be taken carefully and would benefit from comparisons using other methodologies.

Additionally, while we use a large and rich dataset, it is the result of the combination of various data sources and surveys conducted for different reasons. To better inform policymakers on effective policies to enable greater digital payment acceptance and usage, longitudinal data collection and analysis are critical to identify the effectiveness of various public and private sector initiatives. Unfortunately, we lack a time-series component to study how such incentives impact acceptance and usage over time within a country. Furthermore, more standardized cross-country merchant surveys should be encouraged to better understand why certain incentives work better in some countries than others. Such data would allow better estimation of impact of an initiative within a country which is likely to be more important to base policy upon.

Finally, we would encourage policymakers to consider the role of nonbanks in the provision of payment services to increase access to digital payment services while adopting proper safeguards to ensure a safe and efficient payment system with adequate end-user protections especially when a large proportion of the population is unbanked. Technological advances often brought to the marketplace by nonbanks are likely to increase digital connectivity among consumers and businesses and increase the acceptance and usage of digital payments.

## Supporting information

S1 Appendix The data. (DOCX)

S2 Appendix Robustness checks [59–61]. (DOCX)
