# Supplementary material for: Assessing incentives to increase digital payment acceptance and usage: A machine learning approach
Source: PLoS One. 2022 Nov 2;17(11):e0276203. doi: 10.1371/journal.pone.0276203 (PMC9629583; doi:10.1371/journal.pone.0276203)
Supplement: S1 Appendix — (DOCX) [file pone.0276203.s001.docx]

**Appendix A1: The Data**

**List of countries in the country-level sample:** Afghanistan, Albania, Angola, Argentina, Armenia, Australia, Austria, Azerbaijan, Bangladesh, Belgium, Bhutan, Bolivia, Bosnia and Herzegovina, Botswana, Brazil, Bulgaria, Burundi, Cambodia, Canada, Central African Republic, Chile, China, Colombia, Costa Rica, Croatia, Denmark, Dominican Republic, Ecuador, Egypt, El Salvador, Estonia, Finland, France, Georgia, Germany, Greece, Guatemala, Haiti, Honduras, Hong Kong SAR, China, Hungary, India, Indonesia, Iran, Iraq, Israel, Italy, Jamaica, Japan, Jordan, Kazakhstan, Kenya, Kuwait, Kyrgyz Republic, Latvia, Lebanon, Lesotho, Liberia, Lithuania, Luxembourg, Madagascar, Malawi, Malaysia, Malta, Mauritania, Mauritius, Mexico, Moldova, Montenegro, Morocco, Myanmar, Namibia, the Netherlands, New Zealand, Nigeria, Norway, Oman, Pakistan, Panama, Paraguay, Peru, Philippines, Poland, Portugal, Russia, Rwanda, Saudi Arabia, Serbia, Slovak Republic, South Africa, Korea, Spain, Sri Lanka, Sudan, Sweden, Tajikistan, Thailand, Tunisia, Turkey, Uganda, Ukraine, the United Kingdom, Uruguay, Vietnam, Zambia, and Zimbabwe.

**List of countries in the merchant-level sample:** Colombia, France, Kenya, Lithuania, Morocco, Pakistan, and Turkey

**Variables from the country-level sample**

| **Variable** | **Definition** | **Source and year** | **Mean** | **St. dev** |
| --- | --- | --- | --- | --- |
| account | Account (% age 15+) | WBG Findex, 2014 | 0.63 | 0.25 |
| agents_psp_pc | Agents of payment service providers per 100,000 adults | WBG GPSS, 2014 | 2,58 | 2,75 |
| aml_cft_risks | Are AML/CFT risks assessed during authorization process for modified or new financial products? | WBG FICP, 2017 | 0,93 | 0,24 |
| atms_pc | Number of ATMs per 100,000 adults | IMF FAS, 2014 | 4,29 | 2,25 |
| branches_pc | Number of commercial bank branches per 100,000 adults | IMF FAS, 2014 | 3,13 | 3,50 |
| consumer_risks | Are consumer risks assessed during authorization process for modified or new financial products? | WBG FICP, 2017 | 0,90 | 0,29 |
| credit_card | Credit card ownership (% age 15+) | WBG Findex, 2014 | 0,20 | 0,21 |
| credit_cards_pc | Number of credit cards per 1,000 adults | IMF FAS, 2014 | 0,58 | 0,38 |
| debit_card | Debit card ownership (% age 15+) | WBG Findex, 2014 | 0,43 | 0,32 |
| debit_cards_pc | Number of debit cards per 1,000 adults | IMF FAS, 2014 | 0,61 | 0,44 |
| deposit | Deposit in the past year (% with a financial institution account, age 15+) | WBG Findex, 2014 | 0,73 | 0,15 |
| deposit_accounts_pc | Number of deposit accounts with commercial banks per 1,000 adults | IMF FAS, 2014 | 0,78 | 0,84 |
| emoney_accounts_pc | E-money accounts per 1,000 adults | WBG GPSS, 2014 | 0,10 | 0,28 |
| fi_account | Financial institution account (% age 15+) | WBG Findex, 2014 | 0,61 | 0,27 |
| if_action | Authorities have taken actions, or are considering taking action to address interchange fees | WBG GPSS, 2014 | 0,80 | 0,40 |
| if_high | Authorities consider interchange fees prevailing in the card industry to be high | WBG GPSS, 2014 | 0,57 | 0,49 |
| internet_bill_pay | Used the internet to pay bills in the past year (% age 15+) | WBG Findex, 2014 | 0,23 | 0,24 |
| itu_idi | ITU ICT Development Index | ITU | 0,44 | 0,37 |
| made_received_dig_pay | Made or received digital payments in the past year (% age 15+) | WBG Findex, 2014 | 0,56 | 0,27 |
| main_withdrawal_atm | Main mode of withdrawal: ATM (% with a financial institution account, age 15+) | WBG Findex, 2014 | 0,55 | 0,22 |
| main_withdrawal_teller | Main mode of withdrawal: bank teller (% with a financial institution account, age 15+) | WBG Findex, 2014 | 0,38 | 0,21 |
| max_cost_account | Is the maximum cost to open a savings/current account regulated in this country? | WBG FICP, 2017 | 0,04 | 0,21 |
| mm_account | Mobile money account (% age 15+) | WBG Findex, 2014 | 0,08 | 0,12 |
| mm_accounts_pc | Number of registered mobile money accounts per 1,000 adults | IMF FAS, 2014 | 0,63 | 0,31 |
| mm_agents_pc | Number of registered mobile money agent outlets per 100,000 adults | IMF FAS, 2014 | 1,23 | 1,04 |
| mobile_internet_access_fi | Used a mobile phone or the internet to access a financial institution account in the past year (% age 15+) | WBG Findex, 2014 | 0,22 | 0,23 |
| national_id | Has a national identity card (% age 15+) | WBG Findex, 2014 | 0,91 | 0,122 |
| nfcs | Has a national financial capability/literacy/education strategy (NFCS/NFLS/NFES) already been launched? | WBG FICP, 2017 | 0,04 | 0,21 |
| nfis | Has a national financial inclusion strategy (NFIS) already been launched? | WBG FICP, 2017 | 0,28 | 0,45 |
| nms | Has a national microfinance strategy (NMS) already been launched? | WBG FICP, 2017 | 0,22 | 0,42 |
| no_account_distance | No account because financial institutions are too far away (% age 15+) | WBG Findex, 2014 | 0,09 | 0,09 |
| no_account_documentation | No account because of lack of necessary documentation (% age 15+) | WBG Findex, 2014 | 0,10 | 0,09 |
| no_account_expensive | No account because financial services are too expensive (% age 15+) | WBG Findex, 2014 | 0,15 | 0,11 |
| no_account_family | No account because someone in the family has an account (% age 15+) | WBG Findex, 2014 | 0,11 | 0,04 |
| no_account_funds | No account because of insufficient funds (% age 15+) | WBG Findex, 2014 | 0,29 | 0,19 |
| no_account_religion | No account because of religious reasons (% age 15+) | WBG Findex, 2014 | 0,03 | 0,03 |
| no_account_trust | No account because of lack of trust in financial institutions (% age 15+) | WBG Findex, 2014 | 0,09 | 0,07 |
| operational_risks | Are operational risks assessed during authorization process for modified or new financial products? | WBG FICP, 2017 | 0,95 | 0,21 |
| pension_fi | Received a public sector pension: into a financial institution account (% age 15+) | WBG Findex, 2014 | 0,12 | 0,05 |
| pos_pc | POS terminals per 100,000 adults | WBG GPSS, 2014 | 614 | 994 |
| school_fees | Paid school fees in the past year (% age 15+) | WBG Findex, 2014 | 0,32 | 0,24 |
| sent_received_dom_remit | Sent or received domestic remittances in the past year (% age 15+) | WBG Findex, 2014 | 0,30 | 0,11 |
| tax_incentives_savings | Are tax incentive savings schemes in place to promote financial inclusion? | WBG FICP, 2017 | 0,24 | 0,43 |
| wages | Received wages in the past year (% age 15+) | WBG Findex, 2014 | 0,37 | 0,15 |
| wages_fi | Received wages: into a financial institution account (% age 15+) | WBG Findex, 2014 | 0,25 | 0,19 |
| withdrawal | Withdrawal in the past year (% with a financial institution account, age 15+) | WBG Findex, 2014 | 0,75 | 0,16 |
| m_fiscal_incent_long | Merchant fiscal incentive implemented long time ago | Authors’ own elaboration, 2014 | 0.25 | 0,37 |
| m_fiscal_incent_recent | Merchant fiscal incentive implemented recently | Authors’ own elaboration, 2014 | 0,22 | 0,44 |
| c_fiscal_incent_long | Consumer fiscal incentives (VAT reductions, income tax reductions) long time ago | Authors’ own elaboration, 2014 | 0,37 | 0,21 |
| c_fiscal_incent_recent | Consumer fiscal incentives (VAT reductions, income tax reductions) recently | Authors’ own elaboration, 2014 | 0,44 | 0,29 |
| lott_long | Lotteries implemented long-time ago | Authors’ own elaboration, 2014 | 0,18 | 0,19 |
| lott_recent | Lotteries implemented recently | Authors’ own elaboration, 2014 | 0,02 | 0,17 |
| mandated_epa_long | Mandated acceptance of electronic payments implemented long time ago | Authors’ own elaboration, 2014 | 0,25 | 0,27 |
| mandated_epa_recent | Mandated acceptance of electronic payments implemented recently | Authors’ own elaboration, 2014 | 0,22 | 0,31 |
| subsid_POS_long | Subsidized POS terminals implemented long-time ago | Authors’ own elaboration, 2014 | 0,17 | 0,19 |
| subsid_POS_recent | Subsidized POS terminals implemented recently | Authors’ own elaboration, 2014 | 0,15 | 0,18 |
| cash_limits_long | Cash transaction limits (or significant cash disincentives) implemented long time ago | Authors’ own elaboration, 2014 | 0,32 | 0,37 |
| cash_limits_recent | Cash transaction limits (or significant cash disincentives) implemented recently | Authors’ own elaboration, 2014 | 0,38 | 0,53 |
| killer_app | A "killer app" exists for mobile payment adoption | Authors’ own elaboration, 2014 | 0,12 | 0,09 |
| m_fees_reduced | Lowering of merchant fees | Authors’ own elaboration, 2014 | 0,39 | 0,17 |
| crime | Serious assaults per 100,000 population | United Nations Office for Drug and Crime (UNDOC), 2014 | 112 | 160 |
| shadow_econ | Shadow economy over GDP | Authors’ own estimations, 2014 | 28,12 | 14,55 |

**Variables from the merchant-level sample**

| **Variable** | **Definition** | **Units** | **Mean** | **St. dev** |
| --- | --- | --- | --- | --- |
| repeat_customers | Repeat customers | Proportion of customers | 0.66 | 0.09 |
| transactions_pd | Transactions per day | Gross volume | 209.86 | 321.25 |
| transactions_pa | Transactions per annum | Gross volume | 71993.80 | 110484.84 |
| cash_val | Value of P2B cash payments | Proportion of all P2B payments | 0.60 | 0.32 |
| cheque_val | Value of P2B cheque payments | Proportion of all P2B payments | 0.04 | 0.11 |
| transfer_val | Value of P2B bank transfers | Proportion of all P2B payments | 0.02 | 0.08 |
| mobile_val | Value of P2B mobile transfers | Proportion of all P2B payments | 0.02 | 0.07 |
| cards_val | Value of P2B card payments | Proportion of all P2B payments | 0.31 | 0.31 |
| cash_vol | Volume of P2B cash payments | Proportion of all P2B payments | 0.65 | 0.30 |
| cheque_vol | Volume of P2B cheque payments | Proportion of all P2B payments | 0.04 | 0.11 |
| transfer_vol | Volume of P2B bank transfers | Proportion of all P2B payments | 0.02 | 0.07 |
| mobile_vol | Volume of P2B mobile transfers | Proportion of all P2B payments | 0.02 | 0.07 |
| cards_vol | Volume of P2B card payments | Proportion of all P2B payments | 0.27 | 0.28 |
| epay_val | Value of P2B electronic payments | Proportion of all P2B payments | 0.36 | 0.31 |
| epay_vol | Volume of P2B electronic payments | Proportion of all P2B payments | 0.31 | 0.28 |
| c_paper_pref | Customers prefer to pay with paper-based methods? | Binary (0 - No / 1 - Yes) | 0.52 | 0.50 |
| m_paper_pref | Merchant prefers paper-based payment methods? | Binary (0 - No / 1 - Yes) | 0.58 | 0.49 |
| pos | Merchant has a POS terminal? | Binary (0 - No / 1 - Yes) | 0.68 | 0.47 |
| bank_account | Merchant has a bank account? | Binary (0 - No / 1 - Yes) | 0.93 | 0.26 |
| epay | Electronic payments at retailer? (Yes/No) | Binary (0 - No / 1 - Yes) | 0.73 | 0.44 |
| region | Region |  | 1.75 | 0.81 |
| group | Income Group |  | 0.48 | 0.50 |
| grocery | Grocery? | Binary (0 - No / 1 - Yes) | 12.56 | 13.45 |
| type | Type of retailer |  | 0.85 | 0.36 |
| years_service | Years in business |  | 0.87 | 0.33 |
| formal | Formal? | Binary (0 - No / 1 - Yes) | 0.44 | 0.26 |
| urban | Urban? | Binary (0 - No / 1 - Yes) | 0.17 | 0.22 |
| location | City/region. etc. |  | 0.28 | 0.45 |
| branches | Number of Retailer Branches |  | 28.05 | 2.86 |
| num_employees | Number of Employees |  | 3.54 | 1.47 |
| female_owned | Female owned? | Binary (0 - No / 1 - Yes) | 0.28 | 0.19 |
| days_open_month | Days open per month |  | 28.2 | 2.86 |
| consumer_income | Income demographic of customers |  | 3.54 | 1.47 |
| epay_val_country | Value of P2B MSME e-payments in the country | Proportion of all P2B payments at MSMEs | 0.27 | 0.18 |
| epay_vol_country | Volume of P2B MSME e-payments in the country | Proportion of all P2B payments at MSMEs | 0.19 | 017 |
| avg_num_suppliers_pw | Average number of suppliers per week |  | 13.98 | 22.05 |
| avg_num_supplier_pmts_pm | Average number of supplier payments per month |  | 21.77 | 31.20 |
| avg_num_supplier_pmts_pa | Average number of supplier payments per annum |  | 261.34 | 374.85 |
| cash_val_b2b | Value of B2B cash payments | Proportion of all B2B payments | 0.32 | 0.39 |
| cheque_val_b2b | Value of B2B cheque payments | Proportion of all B2B payments | 0.23 | 0.32 |
| transfer_val_b2b | Value of B2B bank transfers | Proportion of all B2B payments | 0.36 | 0.40 |
| mobile_val_b2b | Value of B2B mobile transfers | Proportion of all B2B payments | 0.01 | 0.04 |
| cards_val_b2b | Value of B2B card payments | Proportion of all B2B payments | 0.01 | 0.08 |
| cash_vol_b2b | Volume of B2B cash payments | Proportion of all B2B payments | 0.33 | 0.39 |
| cheque_vol_b2b | Volume of B2B cheque payments | Proportion of all B2B payments | 0.23 | 0.33 |
| transfer_vol_b2b | Volume of B2B bank transfers | Proportion of all B2B payments | 0.34 | 0.39 |
| mobile_vol_b2b | Volume of B2B mobile transfers | Proportion of all B2B payments | 0.01 | 0.03 |
| cards_vol_b2b | Volume of B2B card payments | Proportion of all B2B payments | 0.01 | 0.07 |
| epay_val_b2b | Value of B2B electronic payments | Proportion of all B2B payments | 0.44 | 0.41 |
| epay_vol_b2b | Volume of B2B electronic payments | Proportion of all B2B payments | 0.41 | 0.41 |
| employee_pmts_pm | Number of employee payments per month |  | 1.40 | 0.81 |
| employee_pmnts_pa | Number of employee payments per annum |  | 282,02 | 413,03 |
| cash_val_b2p | Value of B2P cash payments | Proportion of all B2P payments | 0.41 | 0.46 |
| cheque_val_b2p | Value of B2P cheque payments | Proportion of all B2P payments | 0.08 | 0.23 |
| transfer_val_b2p | Value of B2P bank transfers | Proportion of all B2P payments | 0.50 | 0.47 |
| mobile_val_b2p | Value of B2P mobile transfers | Proportion of all B2P payments | 0.01 | 0.04 |
| cards_val_b2p | Value of B2P card payments | Proportion of all B2P payments | 0.01 | 0.03 |
| epay_val_b2p | Value of B2P electronic payments | Proportion of all B2P payments | 0.50 | 0.47 |

Additionally, in each one of the exercises (country-level and merchant-level) we use variables from the other one. In the country-level exercise, for example, we use the share of digital payments made to MSMRs by individuals as a dependent variable but also as an explanatory variable when POS is the dependent variable. Similarly, we use the share of B2B and BTP digital payments and we also use a breakdown of these variables for grocery and non-grocery establishments (as estimated by Euromonitor from merchant data and then extrapolated to a country-level database).
