# Supplementary material for: Assessing incentives to increase digital payment acceptance and usage: A machine learning approach
Source: PLoS One. 2022 Nov 2;17(11):e0276203. doi: 10.1371/journal.pone.0276203 (PMC9629583; doi:10.1371/journal.pone.0276203)
Supplement: S2 Appendix — (DOCX) [file pone.0276203.s002.docx]

**Appendix A2: Robustness Checks**

As for alternative models, comparisons can be made with parametric standard approaches or with other machine learning algorithms. As for parametric model, a natural alternative would be running an ordered logit model, as the dependent variables take the values 1, 2, 3, 4 depending on the level of POS adoption or the share of electronic P2B payments. This also involves considering three constant cuts to evaluate the estimation at the four levels. As our benchmark model is the country-level sample, we run the logit model with the same set of variables used in the machine learning methods are employed. The results are shown in Table A2.1. Only the most relevant variables are shown although the 23 indicators reported in the earlier section are included. The findings are in line with those of the random and causal forests. Alternative specifications included different combinations of some of the most relevant (incentive-related) variables excluding others to study their effect both with and without other influences controlled for. The results were in line to those shown in the table.

| **Table A2.1 Ordered logit estimation results** | | | | |
| --- | --- | --- | --- | --- |
| **Dependent variable** | **POS adoption** | | **Share of P2B electronic payments** | |
|  | Coeff. | Std. error | Coeff. | Std. error |
| Wages paid into a transaction account | 0.0283*** | 0.009 | 0.0418** | 0.018 |
| Share of P2B electronic payments | 0.0357** | 0.016 | - | - |
| Information and Communication Technologies (ICT) | 0.1205** | 0.049 | 0.1933*** | 0.044 |
| Account ownership | 0.0742* | 0.041 | - | - |
| National ID | 0.0205** | 0.010 | - | - |
| National financial strategy exists | 0.0928*** | 0.033 | - | - |
| Shadow Economy | -0.0089** | -0.004 | - | - |
| Share of B2B electronic payments | - | - | 0.0419*** | 0.015 |
| Previous card penetration (2011) | - | - | 0.0083* | 0.048 |
| POS adoption | - | - | 0.0902*** | 0.029 |
| Agents of payment services providers | - | - | 0.0351*** | 0.010 |
| Constant cut1 | 0.0362*** | 0,008 | 0.0403*** | 0.009 |
| Constant cut2 | 1.1929*** | 0,382 | 1.0023*** | 0.363 |
| Constant cut3 | 2.9402*** | 0.928 | 3.1987*** | 0.103 |
| Other controls | Yes | Yes | Yes | Yes |
| Errors clustered at the country level | Yes | Yes | Yes | Yes |
| Observations | 8,586 | | 8,586 | |
| Pseudo R^2^ | 0.4830 | | 0.5775 | |
| Log Likelihood | -1512.19 | | -1728.30 | |
| Note: *** p<0.01, ** p<0.05, * p<0.1 | | | | |

We also benchmark our results to those of two alternative machine learning algorithms: extreme gradient boosting and Bayesian networks. Gradient boosting is based on the idea of whether a weak learner can be modified to become better. As [59] argues, the weak learning method is used several times to get a succession of hypotheses, each iteration is refocused on the examples that the previous ones found difficult and misclassified. Then, using a training sample (y, x) the goal of the algorithm is to obtain an estimate of the function F(x) that minimizes the expected value of a loss function over the joint distribution of all the observed values. Among the gradient boosting methods used in practice, Extreme Gradient Boosting, is widely used due to its efficiency. Compared to other gradient boosting methods, extreme gradient boosting uses a more regularized model formalization to control over-fitting [60].

The second alternative, the Bayesian network, is a direct acyclic graph encoding assumptions of conditional independence. The nodes are stochastic variables, and the arcs show the dependency between the nodes. A Bayesian network is defined by a finite set N = {A,B,...} of nodes (vertices), a set L of arcs (edges) and a joint probability density function. In this sense, a Bayesian network classifier is simply a Bayesian network applied to classification, that is, the prediction of the probability of some discrete (class) variable Y given some features X [61]. When we apply both extreme gradient boosting or Bayesian networks, we identify that the most relevant factors that explain POS adoption and the share of P2B electronic payments coincide with those identified by random forests. However, as shown in Table A2-2, when we use a subsample of data (30%) to check the out-of-sample accuracy of the predictions, the random forest outperforms the alternative machine learning algorithms and the logit model.

| **Table A2.2: Alternative Model Performance in terms of Predictive Accuracy** |
| --- |

|  |  | **Out-of-sample accuracy (70/30% split)** | |
| --- | --- | --- | --- |
|  |  | POS adoption | Share of P2B electronic payments |
| **Random forest** | | 89.91% | 92.14% |
| **Extreme Gradient Boosting** | | 80.17% | 82.29% |
| **Bayesian Networks (Naive Bayes)** | | 59.94% | 42.27% |
| **Logit** | | 76.12% | 58.15% |

| **Table A2.3: Random Forest Hyperparameters and Cross-Validation of the Algorithm** | | | |
| --- | --- | --- | --- |
|  | **Panel A. Random Forest Hyperparameters** | |  |
|  | POS adoption | Share of P2B electronic payments |  |
| **Number of Trees** | 1,000 | 1,000 |  |
| **Number of Features for each Tree** | 9 | 12 |  |
| **Maximum Depth of the Tree** | 20 | 20 |  |
|  | **Panel B. Cross-validation accuracy** | |  |
| **K-fold cross validation** | 88.31% | 71.88% |  |
| **Repeated K-fold cross-validation** | 89.04% | 73.35% |  |

Additionally, as the random forest require selecting some hyperparameters (i.e., number of features for each tree), which are tuned to obtain the optimal parameter values for higher accuracy. The performance of all machine learning methods is computed after optimizing the hyper-parameters for each method. We employed the following R packages: tune, caret, tuneRF and xgboost. Finally, in order to check the stability of the accuracy of the results, we employ two cross validation methods: the k-fold cross-validation and the repeated K-fold cross-validation. In doing so, the dataset is split into 10 groups (k=10), since this value has been shown empirically to yield test error rate estimates that suffer neither from excessively high bias nor from very high variance. In case of the repeated K-fold cross-validation, the data is split into 10-folds, repeating the process five times. The results reported in Table A2.3 confirm the validity of the random forest model.
